# Immune checkpoint activity regulates polycystic kidney disease progression

**DOI:** 10.1172/jci.insight.161318

**Published:** 2023-06-22

**Authors:** Emily K. Kleczko, Dustin T. Nguyen, Kenneth H. Marsh, Colin D. Bauer, Amy S. Li, Marie-Louise T. Monaghan, Michael D. Berger, Seth B. Furgeson, Berenice Y. Gitomer, Michel B. Chonchol, Eric T. Clambey, Kurt A. Zimmerman, Raphael A. Nemenoff, Katharina Hopp

**Affiliations:** 1 Department of Medicine, Division of Renal Diseases and Hypertension,; 2 Consortium for Fibrosis Research and Translation, and; 3 Department of Anesthesiology, University of Colorado Anschutz Medical Campus, Aurora, Colorado, USA.; 4 Department of Internal Medicine, Division of Nephrology, University of Oklahoma Health Sciences Center, Oklahoma City, Oklahoma, USA.

**Keywords:** Nephrology, Adaptive immunity, Monogenic diseases

## Abstract

Innate and adaptive immune cells modulate the severity of autosomal dominant polycystic kidney disease (ADPKD), a common kidney disease with inadequate treatment options. ADPKD has parallels with cancer, in which immune checkpoint inhibitors have been shown to reactivate CD8^+^ T cells and slow tumor growth. We have previously shown that in PKD, CD8^+^ T cell loss worsens disease. This study used orthologous early-onset and adult-onset ADPKD models (*Pkd1* p.R3277C) to evaluate the role of immune checkpoints in PKD. Flow cytometry of kidney cells showed increased levels of programmed cell death protein 1 (PD-1)/cytotoxic T lymphocyte associated protein 4 (CTLA-4) on T cells and programmed cell death ligand 1 (PD-L1)/CD80 on macrophages and epithelial cells in *Pkd1*^RC/RC^ mice versus WT, paralleling disease severity. PD-L1/CD80 was also upregulated in ADPKD human cells and patient kidney tissue versus controls. Genetic PD-L1 loss or treatment with an anti–PD-1 antibody did not impact PKD severity in early-onset or adult-onset ADPKD models. However, treatment with anti–PD-1 plus anti–CTLA-4, blocking 2 immune checkpoints, improved PKD outcomes in adult-onset ADPKD mice; neither monotherapy altered PKD severity. Combination therapy resulted in increased kidney CD8^+^ T cell numbers/activation and decreased kidney regulatory T cell numbers correlative with PKD severity. Together, our data suggest that immune checkpoint activation is an important feature of and potential novel therapeutic target in ADPKD.

## Graphical abstract

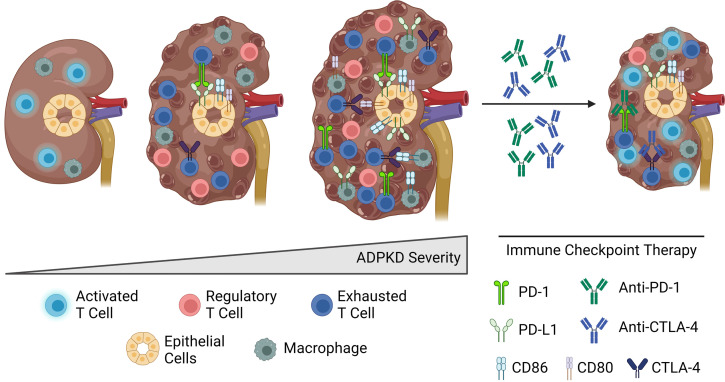

## Introduction

Autosomal dominant polycystic kidney disease (ADPKD), caused predominantly by mutations to *PKD1* or *PKD2*, is the most common monogenic kidney disease (1). It is characterized by the growth of bilateral kidney cysts leading to end-stage kidney disease in 50% of patients by middle age (1). Patients with ADPKD present with substantial variability in disease severity and progression that cannot be solely explained by genic or allelic effects (2–4). Underlying reasons for this phenotypic heterogeneity remain poorly understood but likely involve differences in the cystic microenvironment (CME). In cancer, a disease that shares multiple characteristics with PKD, innate and adaptive immune cells within the tumor microenvironment significantly modulate disease progression (5, 6). Similarly, studies of the CME suggest that immune cells play a key role in modulating PKD severity (7). Multiple groups have demonstrated that kidney macrophages drive cyst expansion in mouse models of ADPKD (7–9). Further, urinary CD4^+^ T cell numbers were reported to correlate with renal function loss in patients with ADPKD, and we have shown that, correlative with disease severity, both CD4^+^ and CD8^+^ T cell numbers increase in cystic kidneys of an orthologous ADPKD mouse model and specifically localize to cystic lesions (10, 11). Immunodepletion of CD8^+^ T cells resulted in more rapid cyst growth, suggesting an anti-cystogenic role of these cells (10). The functional role of CD4^+^ T cells has not been studied in the setting of ADPKD and is complicated owing to their complex subtyping with opposing functions (12).

In cancer, tumor-specific naive CD8^+^ T cells become activated by recognizing tumor-specific neoantigens and self-antigens, leading to production of cytotoxic molecules, such as granzymes and perforin, that directly kill tumor cells. Further, they secrete cytokines, such as interferon-γ (IFN-γ), which increase expression of major histocompatibility complex (MHC) class I antigens by tumor cells, thereby rendering them better targets for CD8^+^ T cell–mediated killing (13). A similar immunosurveillance role for CD8^+^ T cells can be proposed in PKD where CD8*^+^* T cell recruitment and activation is a response to neoantigens produced by the dedifferentiation process of the tubular epithelium during cyst development/growth (e.g., genomic instability, somatic mutations, damaged interstitial/epithelial cells). This correlates with our prior findings that CD8^+^ T cells localize to cystic lesions, that levels of IFN-γ are higher in cystic kidneys compared with controls, and that CD8^+^ T cell loss resulted in more rapidly progressive PKD (10).

Despite the presence of tumor-infiltrating effector CD8^+^ T cells, most tumors continue to progress, suggesting that tumor-reactive CD8^+^ T cells become dysfunctional, i.e., exhausted, during tumor progression (14, 15). This has been attributed to tumors being able to engage mechanisms that drive immunosuppression. Immunosuppressive cells include CD4^+^ regulatory T cells (Tregs), which inhibit CD8^+^ T cell activity via direct cytolysis or release of inhibitory cytokines (e.g., TGF-β, IL-10, IL-35), as well as M2-like tumor-associated macrophages (TAMs) and myeloid-derived suppressor cells (MDSCs) (16). Both MDSCs and TAMs produce high levels of immunosuppressive proteins such as IL-10, TGF-β, IDO1, ARG1, iNOS, and immune checkpoint ligands (e.g., programmed cell death ligand 1 [PD-L1]), causing CD8^+^ T cells to undergo metabolic reprogramming, lose effector function (secretion of TNF-α, IL-2, IFN-γ), and sustain expression of immune checkpoint receptors (programmed cell death protein 1 [PD-1], TIM-3, cytotoxic T lymphocyte associated protein 4 [CTLA-4]), all features of exhausted CD8^+^ T cells (13, 16). Interestingly, PKD-associated macrophages have been compared to TAMs because of multiple phenotypic and functional similarities (17). Furthermore, we recently published that an increased number of kidney Tregs are found in the setting of ADPKD and that IDO1 has a functional role in ADPKD pathogenesis (18, 19).

Typically, immune checkpoints, such as PD-1|PD-L1 or CTLA-4|CD80/CD86, are proteins that regulate immune homeostasis, prevent excessive CD8^+^ T cell activation, and protect against autoimmunity (20). Tumors have exploited this pathway to escape immune surveillance. In tumors, PD-1 or CTLA-4 is expressed on CD8^+^ T cells and binds to PD-L1 or CD80/CD86 expressed on tumor cells and tumor-infiltrating myeloid cells (e.g., TAMs), respectively. Interaction of these proteins dampens effector CD8^+^ T cell function and diminishes their ability to recognize and clear tumor cells (20, 21). Immune checkpoint inhibitors (ICIs) are monoclonal antibodies that make up a novel class of immunotherapy drugs that disrupt the interaction between ligand and receptor (e.g., PD-1 and PD-L1), leading to reactivation of antitumor T cells. To date, more than 7 different immune checkpoints of T cell activation have been targeted therapeutically in clinical cancer trials (22). Among these, CTLA-4 and PD-1 have been found to be the most robust in reactivating CD8^+^ T cells. Indeed, ipilimumab, which blocks CTLA-4, was the first immune checkpoint antibody approved by the FDA for treatment of melanoma, and ICIs have become the standard of care in multiple different cancer types, including renal cell carcinoma (23–25).

Different roles for immune checkpoint–associated immunomodulation have been suggested in various kidney pathologies. PD-1–deficient mice develop lupus-like glomerulonephritis as they age, and anti–PD-1 treatment of a murine adriamycin nephropathy model led to worsened glomerular and tubulointerstitial injury, consistent with a role for immune checkpoints in self-tolerance (26, 27). Similarly, adenovirus-driven PD-L1 overexpression improved lupus nephritis–associated pathologies in mice (28). Reduced CTLA-4 expression on circulating CD19^+^ B cells and CD4^+^ or CD8^+^ T cells in patients with primary proliferative and nonproliferative glomerulonephritis versus control participants was suggested to contribute to continuous activation of T cells and pathogenesis (29). Contrastingly, a second study found PD-1 expression to be significantly elevated on peripheral blood CD4^+^ or CD8^+^ T cells and CD19^+^ B cells of patients with primary proliferative and nonproliferative glomerulonephritis patients versus control participants and correlative with disease severity parameters (30). Last, increased PD-L1 expression on tubular epithelial cells was found in kidney biopsies of patients with IgA nephropathy, interstitial nephritis, and lupus nephritis compared with normal kidneys, although the functional relevance remained unclear (31).

Given the similarities between cancer and ADPKD, which has been described as a “neoplasia in disguise,” we examined whether targeting immune checkpoints represents a novel therapeutic target for treatment of ADPKD (5, 32). Using an orthologous model of ADPKD1, the *Pkd1* p.R3277C mouse (33, 34), with varying rates of disease progression, we found upregulation of both PD-1 and CTLA-4 on kidney CD8^+^ T cells as well as PD-L1 and CD80/CD86 on kidney macrophages and epithelial cells. While genetic loss of PD-L1 or inhibition of either PD-1 or CTLA-4 with a monoclonal antibody did not slow cyst progression, combination treatment of anti–PD-1 and anti–CTLA-4 significantly slowed PKD, suggesting, as seen in cancer clinical trials, that dual immune checkpoint blockade increases efficacy and response rate (20, 35). Our data also define adaptive immunity pathways in the CME that are altered in response to these agents.

## Results

### The PD-1|PD-L1 immune checkpoint pathway is induced in an orthologous mouse model of ADPKD.

We analyzed the expression of PD-1 on kidney CD8^+^ T cells as well as PD-L1 on kidney epithelial cells and macrophages using flow cytometry of kidney single-cell suspensions obtained from an orthologous adult-onset ADPKD1 model, the *Pkd1*^RC/RC^ mouse (33). As previously published, the model presents with varying disease course in different strains (10, 33, 34, 36, 37). C57BL/6J *Pkd1*^RC/RC^ mice have gradually progressive PKD with the kidney area occupied by cysts increasing from about 19% at 3 months of age to about 23% at 9 months of age. Comparably, at 3 months of age the cystic kidney area of BALB/cJ *Pkd1*^RC/RC^ mice is about 32% and of 129S6/SvEvTac *Pkd1*^RC/RC^ mice is about 34%, highlighting a more rapid PKD progression (10, 37).

**Figure 1:**
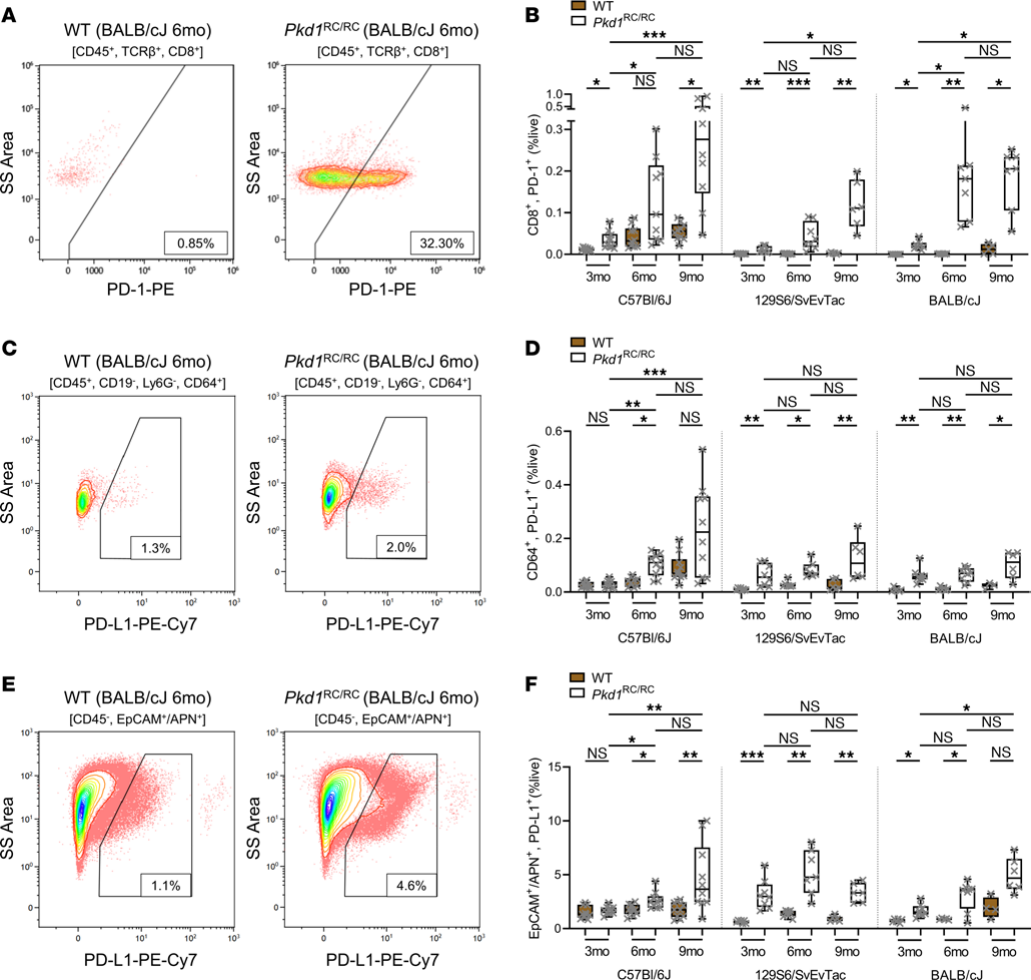
The PD-1|PD-L1 immune checkpoint pathway is induced in an orthologous mouse model of autosomal dominant polycystic kidney disease. Kidneys of WT and *Pkd1*^RC/RC^ mice in the C57BL/6J, 129S6/SvEvTac, and BALB/cJ strains were harvested at 3, 6, and 9 months of age and analyzed by flow cytometry to identify the expression of PD-1 on CD8^+^ T cells (CD45^+^TCRβ^+^CD8^+^; **A** and **B**), PD-L1 on macrophages (CD45^+^CD19^–^Ly6G^–^CD64^+^; **C** and **D**), and PD-L1 on epithelial cells (CD45^–^EpCAM^+^APN^+^; **E** and **F**). (**A**, **C**, and **E**) Representative flow diagrams of 6-month-old BALB/cJ WT and *Pkd1*^RC/RC^ kidneys. SS, side scatter. (**B**, **D**, and **F**) Quantification of PD-1–positive CD8^+^ T cells (**B**), PD-L1–positive CD64^+^ macrophages (**D**), and PD-L1–positive epithelial cells (**F**) as percentage live (%live) comparing WT (brown) with *Pkd1*^RC/RC^ (white) kidneys. PD-1 or PD-L1 expression is significantly upregulated on the respective cell type in *Pkd1*^RC/RC^ compared with WT kidneys and correlative with increasing disease severity in most instances. Data are presented as box plot (25th to 75th percentile and median) with whiskers of 10th and 90th percentiles; single data points are depicted. Kruskal-Wallis 1-way ANOVA with multiple-comparison follow-up by controlling for false discovery rate (Benjamini, Krieger, Yekutieli) was performed. *P < 0.05, **P < 0.01, ***P < 0.001. *N* = 9–10 for C57BL/6J WT or *Pkd1*^RC/RC^, *N* = 6–8 for 129S6/SvEvTac WT or *Pkd1*^RC/RC^, *N* = 5–6 for BALB/cJ WT, and *N* = 7–8 BALB/cJ *Pkd1*^RC/RC^. Data points are half male and half female for all groups.

We examined PD-1|PD-L1 expression in kidneys obtained from *Pkd1*^RC/RC^ mice and strain-matched wild-type (WT) mice, male and female mice combined, at 3, 6, and 9 months of age to correlate the activation of this immune checkpoint with severity of cystic kidney disease. Details on the PKD phenotype parameters of these mice have been previously published (10, 37). In all 3 strains, we found that expression of PD-1 on kidney CD8^+^ T cells in *Pkd1*^RC/RC^ mice increased correlative with cystic kidney disease severity in comparison with strain-, sex-, and age-matched WT mice (Figure 1, A and B). As previously published, C57BL/6J WT mice have more kidney CD8^+^ T cells compared with age- and sex-matched mice in the 129S6/SvEvTac or BALB/cJ strain (10). Correlatively, C57BL/6J WT mice had a high basal level of PD-1–positive CD8^+^ T cells, which increased in *Pkd1*^RC/RC^ mice at all 3 analyzed time points. This increase of PD-1–positive CD8^+^ T cells paralleled the overall increase in CD8^+^ T cell numbers (Supplemental Table 1; supplemental material available online with this article; https://doi.org/10.1172/jci.insight.161318DS1). However, in the strains that present with more rapidly progressive PKD (129S6/SvEvTac or BALB/cJ), not only did PD-1–positive CD8^+^ T cells increase in number with more severe cystic kidney disease when *Pkd1*^RC/RC^ mice were compared with WT mice, but PD-1–positive CD8^+^ T cells became more prominent among the CD8^+^ T cell population (Figure 1, A and B, and Supplemental Table 1).

It has been reported in multiple murine PKD models that the number of kidney macrophages increases in cystic kidneys compared with WT (7). Correlatively, multiple studies have shown that depletion of kidney macrophages slows PKD progression in murine models (7). When evaluating kidney macrophage numbers (CD45^+^CD64^+^ cells) in the *Pkd1*^RC/RC^ model compared with WT, we also found this population to increase in all 3 strains. This correlated with cystic kidney disease severity, and kidneys with the mildest cystic burden (C57BL/6J *Pkd1*^RC/RC^ kidneys at 3 and 6 months of age) showed only a moderate increase in CD64^+^ kidney macrophage numbers compared with WT controls (Supplemental Table 2). Since TAMs have been found to be a prominent driver of immunosuppression and are known to express PD-L1 in cancer, we focused on the expression of PD-L1 on CD64^+^ kidney macrophages (38). We found a significant increase of PD-L1–positive macrophages in *Pkd1*^RC/RC^ mice compared with WT even in moderately cystic kidneys (e.g., 6-month-old C57BL/6J *Pkd1*^RC/RC^ mice). This was further pronounced in the same strain at the 9-month time point and persisted in *Pkd1*^RC/RC^ kidneys obtained from strains with more rapidly progressive PKD (129S6/SvEvTac or BALB/cJ; Figure 1, C and D). Importantly, as we observed for PD-1–positive CD8^+^ T cells, in mice with more severe and more rapidly progressive PKD (129S6/SvEvTac or BALB/cJ *Pkd1*^RC/RC^ mice), the proportion of PD-L1–positive CD64^+^ macrophages increased significantly as a percentage of CD64^+^ cells, both in comparison with WT mice and with disease progression (Supplemental Table 1).

Beyond TAMs, tumor cells are another key cell type expressing PD-L1 (38). Paralleling this finding, we found expression of PD-L1 to be significantly increased on kidney epithelial cells (APN^+^ [ANPEP; proximal tubule, collecting duct] or EpCAM^+^ [distal tubule, loop of Henle, collecting duct]; ref. 39) of *Pkd1*^RC/RC^ mice versus WT in all 3 strains (Figure 1, E and F). That expression did not consistently increase as disease progressed may be explained by an overall decrease of kidney epithelial cells as a proportion of live cells with progressive PKD; in advanced PKD, epithelial cell numbers are likely displaced by fibroblasts, immune cells, etc. (Supplemental Table 2). Hence, it is important to note that, in line with our above-reported findings, the proportion of kidney epithelial cells expressing PD-L1 in *Pkd1*^RC/RC^ kidneys increased as disease advanced and in comparison with WT (Supplemental Table 1).

**Figure 2:**
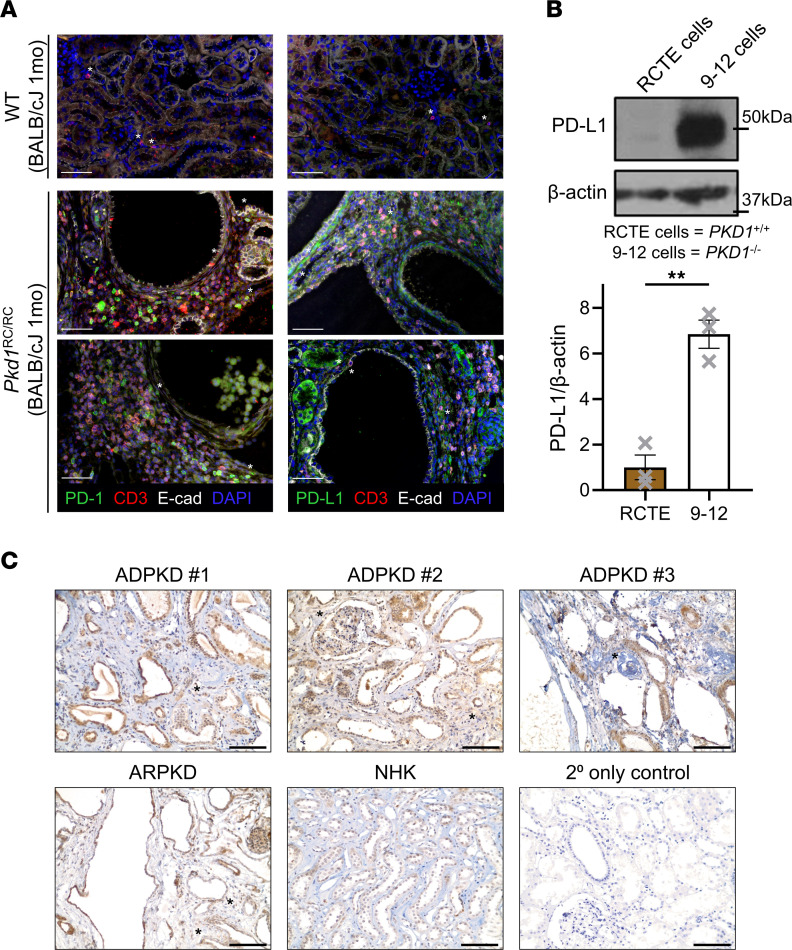
PD-1|PD-L1 immune checkpoint protein expression is increased at cystic lesions of *Pkd1*^RC/RC^ kidneys and in human ADPKD samples. (**A**) Immunofluorescence (IF) labeling of PD-1 (left) and PD-L1 (right) in BALB/cJ WT and *Pkd1*^RC/RC^ kidneys. Merged IF image is shown; Supplemental Figures 1 and 2 contain single-channel images. PD-1 or PD-L1 (green), T cells/CD3 (red), epithelial cells/E-cadherin (white), nuclei/DAPI (blue). In *Pkd1*^RC/RC^ but not WT kidneys, T cells stain positive for PD-1 and are in close proximity to cystic lesions. Further, in cystic regions, interstitial cells and tubular epithelial cells stain positive for PD-L1 in *Pkd1*^RC/RC^ but not WT kidneys. Asterisks in WT: T cells; asterisks in *Pkd1*^RC/RC^: PD-1–positive T cells in contact with epithelial cells, PD-L1–positive interstitial or epithelial cells in contact with T cells. *Pkd1*^RC/RC^: 2 separate animals are shown. Scale bar: 50 μm. (**B**) PD-L1 expression in immortalized human renal cortical tubular epithelial (RCTE) cells (*PKD1^+/+^*) versus 9-12 cells (*PKD1^–/–^*). 9-12 cells have significantly increased levels of PD-L1 compared with control. Top: Representative Western blot image. Bottom: Quantification of Western blots from 3 independent samples. (**C**) IHC staining for PD-L1 in end-stage kidney tissue of 3 ADPKD patients, an autosomal recessive PKD (ARPKD) patient, and a normal human kidney (NHK). 2° only control: kidney tissue slide of an ADPKD patient stained without addition of primary antibody. The tubular/cystic epithelium in ADPKD and ARPKD shows increased expression of PD-L1 compared with tubules of NHK. PD-L1 expression is also found in some interstitial cells within the ADPKD or ARPKD tissue sections (asterisks). Scale bars: 100 μm. Data are presented as mean ± SEM (**B**); single data points are depicted. An unpaired 2-tailed *t* test was performed (**B**). **P < 0.01.

To link our flow cytometry findings to events occurring at the site of cystic lesions, we performed immunofluorescent PD-1 and PD-L1 staining of BALB/cJ WT and *Pkd1*^RC/RC^ kidneys (Figure 2A and Supplemental Figures 1 and 2). As previously published, we observed an enrichment of T cells (CD3^+^) at cystic lesions in *Pkd1*^RC/RC^ kidneys, with few T cells present in WT kidneys (10). Consistent with our flow cytometry findings, in *Pkd1*^RC/RC^ kidneys many T cells surrounding cystic lesions stained positive for PD-1, and a subset were in direct contact with the cystic epithelium (Figure 2A and Supplemental Figure 1). In WT mice, T cells in the kidneys were predominantly negative for PD-1. We also found increased expression of PD-L1 in *Pkd1*^RC/RC^ versus WT kidneys where PD-L1 was nearly undetectable (Figure 2A and Supplemental Figure 2). In *Pkd1*^RC/RC^ kidneys PD-L1 was expressed in 2 distinct patterns: high expression on interstitial cells, i.e., macrophages located close to cystic lesions, and expression on epithelial cells of nondilated or mildly dilated tubules located between larger cysts. In both instances, T cells were found in proximity (Figure 2A and Supplemental Figure 2). Together, these data show that expression of PD-1|PD-L1 is significantly increased on relevant cells and at relevant sites within PKD kidneys of *Pkd1*^RC/RC^ mice compared with WT, suggesting induction of the pathway and an immunosuppressive CME.

### PD-L1 is upregulated in human PKD.

To evaluate whether the immune checkpoint pathway is upregulated in human ADPKD tissue, we performed Western blotting for PD-L1 in immortalized *PKD1^+/+^* cells (renal cortical tubular epithelial [RCTE] cells, WT) and *PKD1^–/–^* cells (9-12 cells, ADPKD). We found minimal PD-L1 expression in RCTE cells. However, 9-12 cells had a significant increase in PD-L1 levels (~7-fold increase) compared with RCTE cells (Figure 2B). Next, we looked for PD-L1 staining in end-stage kidney sections from patients with PKD by immunohistochemistry (IHC). Compared with normal human kidney (NHK) tissue, we found that PD-L1 expression was substantially higher in kidney epithelial cells of ADPKD and autosomal recessive PKD (ARPKD) end-stage kidneys. This increase in expression was predominantly marked in the lining of cysts (Figure 2C, ADPKD #1, ADPKD #2, ARPKD), but also in nondilated tubules, similar to *Pkd1*^RC/RC^ kidneys (Figure 2C, ADPKD #3). In addition, we found positive PD-L1 staining within the cystic interstitium (Figure 2C). These data suggest that this immune checkpoint is engaged in human PKD, mirroring our murine findings.

### Genetic loss of Pd-l1 or immune checkpoint blockade via a monoclonal PD-1–targeting antibody does not ameliorate cystic kidney disease in early- or adult-onset ADPKD.

**Figure 3:**
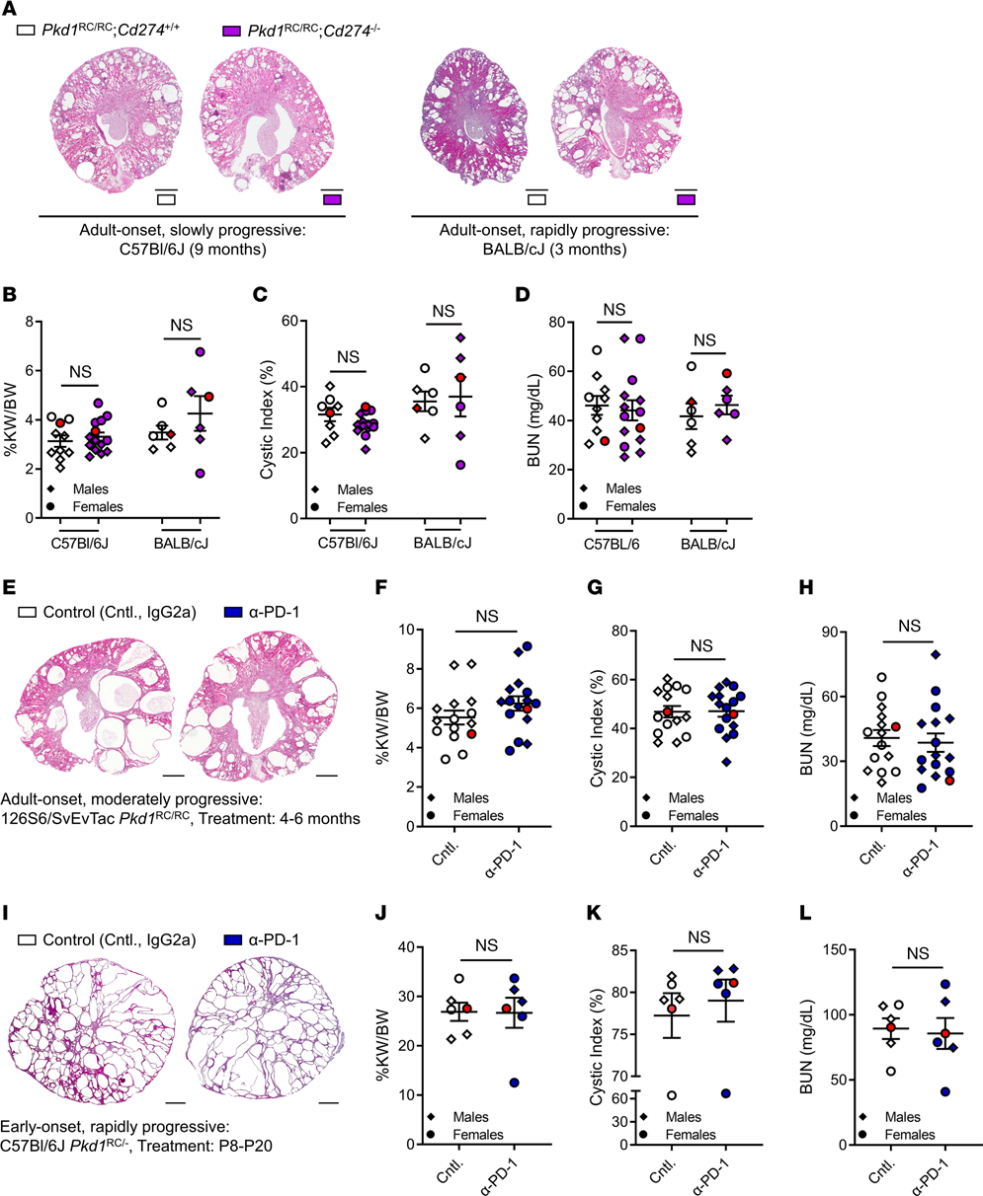
Genetic loss of *Pd-l1* or monoclonal anti–PD-1 treatment does not affect PKD severity in mice with slowly or rapidly progressive ADPKD. (**A**–**D**) *Pkd1*^RC/RC^ mice were crossed with *Pd-l1^–/–^* (NCBI gene ID: *Cd274*) mice to obtain *Pkd1*^RC/RC^ *Cd274^+/+^* (control group; white) and *Pkd1*^RC/RC^ *Cd274^–/–^* (experimental group; purple) mice. Animals were euthanized at 9 months of age in the C57BL/6J strain and 3 months in the BALB/cJ strain. (**E**–**H**) 129S6/SvEvTac *Pkd1*^RC/RC^ mice were treated with 10 mg/kg of anti–PD-1 (α-PD-1; blue) or IgG2a (control, white) twice weekly by i.p. injection from 4 to 6 months of age. (**I**–**L**) C57BL/6J *Pkd1*^RC/–^ mice were treated with 10 mg/kg of α-PD-1 (blue) or IgG2a (control, white) every other day by i.p. injection from P8 until P20. (**A**, **E**, and **I**) Representative H&E cross-sectional images of the kidneys. Scale bars: 1 mm. (**B**, **F**, and **J**) Percentage kidney weight/body weight (%KW/BW). (**C**, **G**, and **K**) Cystic index as measured by kidney area occupied by cysts (%). (**D**, **H**, and **L**) Blood urea nitrogen (BUN) levels. There was no significant difference in any of the analyzed PKD parameters, suggesting that inhibition of the PD-1|PD-L1 immune checkpoint does not impact adult-onset or early-onset PKD progression (also see Supplemental Table 3). Data are presented as mean ± SEM; single data points are depicted. Diamonds, males; circles, females. Red data points indicate the animal shown in **A**, **E**, and **I**. Nonparametric Mann-Whitney tests were performed. (**A**–**D**) *N* = 10–14 for C57BL/6J or *N* = 6 for BALB/cJ mice per group. (**E**–**H**) *N* = 15–16 mice per group. (**I**–**L**) *N* = 6 mice per group.

With the observed induction of PD-1|PD-L1 in our ADPKD1 model compared with WT and an upregulation of PD-L1 in human ADPKD cells and cystic epithelia, we hypothesized that disruption of the PD-1/PD-L1 interaction would ameliorate cystic kidney disease in our orthologous model. To test this, we crossed both the adult-onset C57BL/6J *Pkd1*^RC/RC^ mice with slowly progressive PKD and the adult-onset BALB/cJ *Pkd1*^RC/RC^ mice with rapidly progressive PKD to strain-matched *Pd-l1* (NCBI gene ID: *Cd274*) knockouts and compared PKD-associated phenotypes of *Pkd1*^RC/RC^ *Cd274^+/+^* versus *Pkd1*^RC/RC^ *Cd274^–/–^* mice (Figure 3, A–D). C57BL/6J mice were aged to 9 months of age to ensure we would not miss a possible impact on PKD phenotype at mild to moderate disease stages. BALB/cJ mice were euthanized at 3 months of age, as that time point represents the peak of cystic kidney disease severity after which cyst area and size regress due to cyst collapse and increased fibrotic burden (10, 34, 37). We assessed percentage kidney weight/body weight (%KW/BW), cystic index (kidney cross-sectional area occupied by cysts), cyst size, cyst number, fibrotic index, and kidney function measured as blood urea nitrogen (BUN) levels (Figure 3, B–D, and Supplemental Table 3). Surprisingly, we saw no difference in any of the measured parameters when comparing 9-month-old C57BL/6J *Pkd1*^RC/RC^ *Cd274^+/+^* with *Pkd1*^RC/RC^ *Cd274^–/–^* mice or 3-month-old BALB/cJ *Pkd1*^RC/RC^ *Cd274^+/+^* with *Pkd1*^RC/RC^ *Cd274^–/–^* mice.

Given these unexpected negative results from the genetic ADPKD1/PD-L1 cross, we wanted to assure the robustness of our findings using a second approach — inhibition of the PD-1|PD-L1 pathway via monoclonal antibody therapy. This additional step was especially important as, in the genetic models, the ability to engage the PD-1|PD-L1 immune checkpoint is lost from the time point of conception, potentially triggering compensatory mechanisms (e.g., activation of other immunosuppressive pathways). Here, we chose to use an anti–PD-1 antibody for 3 reasons: (a) meta-analyses of anti–PD-1 versus anti–PD-L1 treatment showed improved efficacy in non–small cell lung cancer (NSCLC) patients receiving anti–PD-1; (b) anti–PD-1 also allows for blockage of the PD-1/PD-L2 interaction, PD-L2 being a second ligand of PD-1, which when highly expressed in solid tumors has been shown to correlate with worse clinical outcome; and (c) anti–PD-1 inhibition complemented our genetic approach by targeting the receptor versus the ligand (40, 41).

First, we treated 4-month-old 129S6/SvEvTac *Pkd1*^RC/RC^ mice for 2 months, twice weekly, by intraperitoneal (i.p.) injection with either 10 mg/kg IgG2a (control) or 10 mg/kg anti–PD-1. Using the 129S6/SvEvTac *Pkd1*^RC/RC^ model allowed us to test the therapeutic efficacy of PD-1|PD-L1 inhibition in a second adult-onset model with more rapidly progressive disease, complementing the BALB/cJ genetic studies. Second, we treated C57BL/6J *Pkd1*^RC/–^ mice with either 10 mg/kg IgG2a (control) or 10 mg/kg anti–PD-1 injected i.p. every other day from postnatal day (P) 8 until P20. C57BL/6J *Pkd1*^RC/–^ mice present with embryonic kidney cysts that rapidly advance, resulting in death by about P25 (33, 34). Hence, this model allowed us to test the therapeutic efficacy of immune checkpoint blockade in an early-onset, rapidly progressive ADPKD model. In both instances we analyzed %KW/BW, cystic index, cyst size, cyst number, fibrotic index, and BUN at treatment endpoint. Again, we saw no difference in the analyzed PKD parameters (Figure 3, E–L, and Supplemental Table 3). Taken together, these data indicate that genetic loss of *Pd-l1* or anti–PD-1 intervention has no effect on kidney cyst growth in our models, despite our data supporting a role for the PD-1|PD-L1 pathway in ADPKD.

### A combination strategy to augment ICI efficacy leads to decreased PKD severity in the BALB/cJ Pkd1^RC/RC^ model.

Multiple clinical trials conducted to test the safety and efficacy of combining anti–PD-1 with anti–CTLA-4 have shown a remarkable increase in response rate and median survival time in multiple cancers, including melanoma, renal cell carcinoma, NSCLC, and hepatocellular carcinoma, resulting in approval of the ipilimumab and nivolumab (anti–PD-1) combination for their treatment (20, 35, 42, 43). Of critical importance, combination treatment showed increased response rates in patients who did not benefit from monotherapy (20, 35).

**Figure 4:**
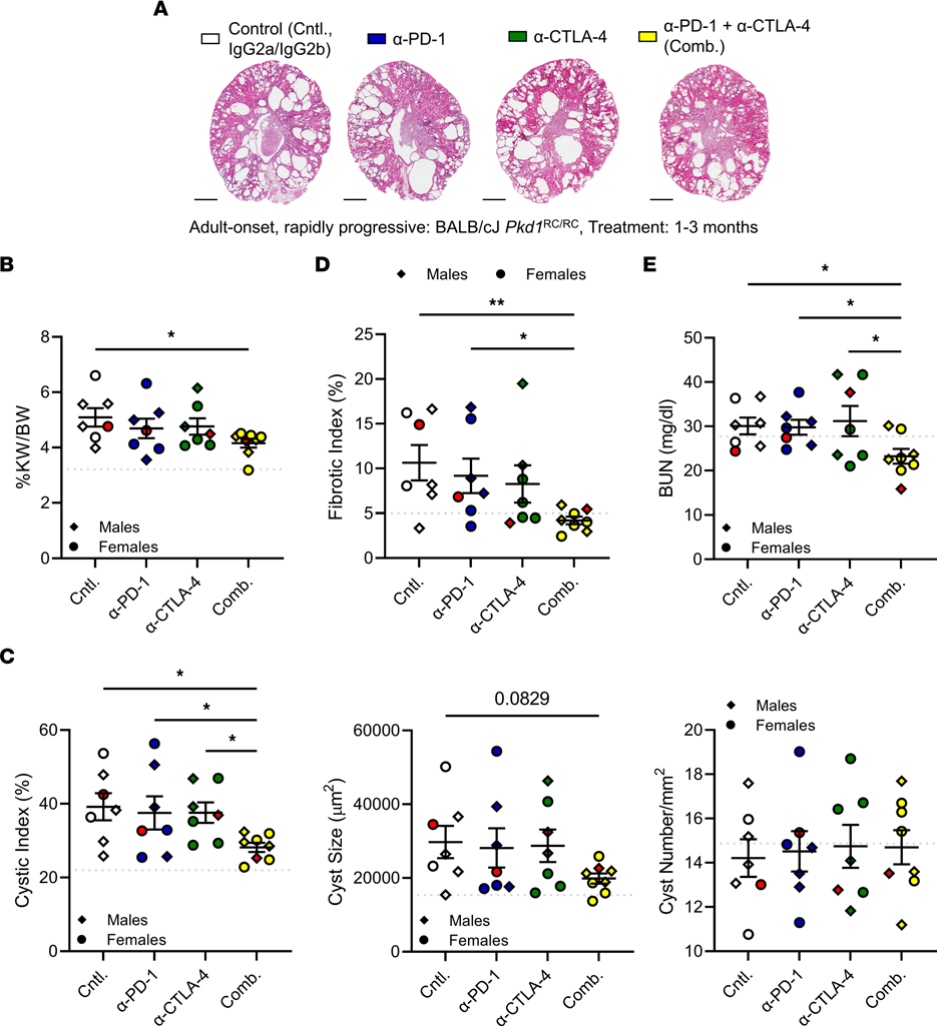
Combination immune checkpoint inhibition reduces cystic kidney disease in *Pkd1*^RC/RC^ mice. BALB/cJ *Pkd1*^RC/RC^ mice were treated from 1 to 3 months of age. One-month baseline PKD phenotypes obtained from a separate cohort of BALB/cJ *Pkd1*^RC/RC^ mice are summarized in Supplemental Table 4 and shown as light gray dotted lines in **B**–**E** (line is set at the analysis mean). Experimental groups: control (white, IgG2a plus IgG2b), α-PD-1 (blue, plus IgG2b), α-CTLA-4 (green, plus IgG2a), and combination (Comb., yellow, α-PD-1 plus α-CTLA-4). (**A**) Representative H&E cross-sectional images of the kidneys. Scale bars: 1 mm. (**B**) Percentage kidney weight/body weight (%KW/BW). (**C**) Cystic index as measured by kidney area occupied by cysts (%), average cyst size, and average cyst number normalized by tissue area. (**D**) Fibrotic index. (**E**) BUN levels. Data are presented as mean ± SEM; single data points are depicted. Diamonds, males; circles, females. Red data points indicate the animal shown in **A**. Kruskal-Wallis 1-way ANOVA with multiple-comparison follow-up by controlling for false discovery rate (Benjamini, Krieger, Yekutieli) was performed. *P < 0.05, **P < 0.01. *N* = 7–8 mice per group. Nonsignificant pairwise comparisons are not shown.

Given the supportive rationale to combine anti–PD-1 and anti–CTLA-4, we tested this approach. We chose the BALB/cJ *Pkd1*^RC/RC^ model for this experiment, as it would allow us to test anti–PD-1 monotherapy in an additional model without repeating the experiments using the 129S6/SvEvTac *Pkd1*^RC/RC^ model. One-month-old BALB/cJ *Pkd1*^RC/RC^ mice (baseline PKD phenotyping characteristics: Supplemental Table 4) were treated twice a week for 8 weeks by i.p. injection with either 10 mg/kg anti–PD-1, 10 mg/kg anti–CTLA-4, the combination of anti–PD-1 and anti–CTLA-4, or control IgG (10 mg/kg IgG2a plus 10 mg/kg IgG2b control). At 3 months of age the animals were euthanized and PKD parameters were analyzed. Like the outcomes observed in the 129S6/SvEvTac *Pkd1*^RC/RC^ model, monotherapy with anti–PD-1 did not alter PKD severity (Figure 4, anti–PD-1 [blue]). Similarly, monotherapy with anti–CTLA-4 did not slow cystic kidney disease growth (Figure 4, anti–CTLA-4 [green]). Remarkably, though, when comparing the animals that received both anti–PD-1 and anti–CTLA-4 with control, we found a significant reduction in nearly all the PKD parameters analyzed: %KW/BW, cystic index, fibrotic index, and BUN (Figure 4). Notably, kidney cyst number did not significantly change, but cyst size showed a trend toward smaller cysts, in mice that received both anti–PD-1 and anti–CTLA-4, suggesting that combination treatment predominantly impacted cyst expansion and not cyst initiation. No health concerns indicative of adverse events were noted in the dually treated mice. Taken together, these data indicate that while monotherapy inhibiting immune checkpoint signaling does not slow PKD progression, suggesting some redundancy in the system, dual immune checkpoint inhibition significantly slows kidney cyst growth in our orthologous ADPKD model.

### Combination immune checkpoint blockade leads to a more robust increase in kidney CD8^+^ T cell numbers/activation and reduced regulatory CD4^+^ T cell numbers compared with monotherapy alone.

**Figure 5:**
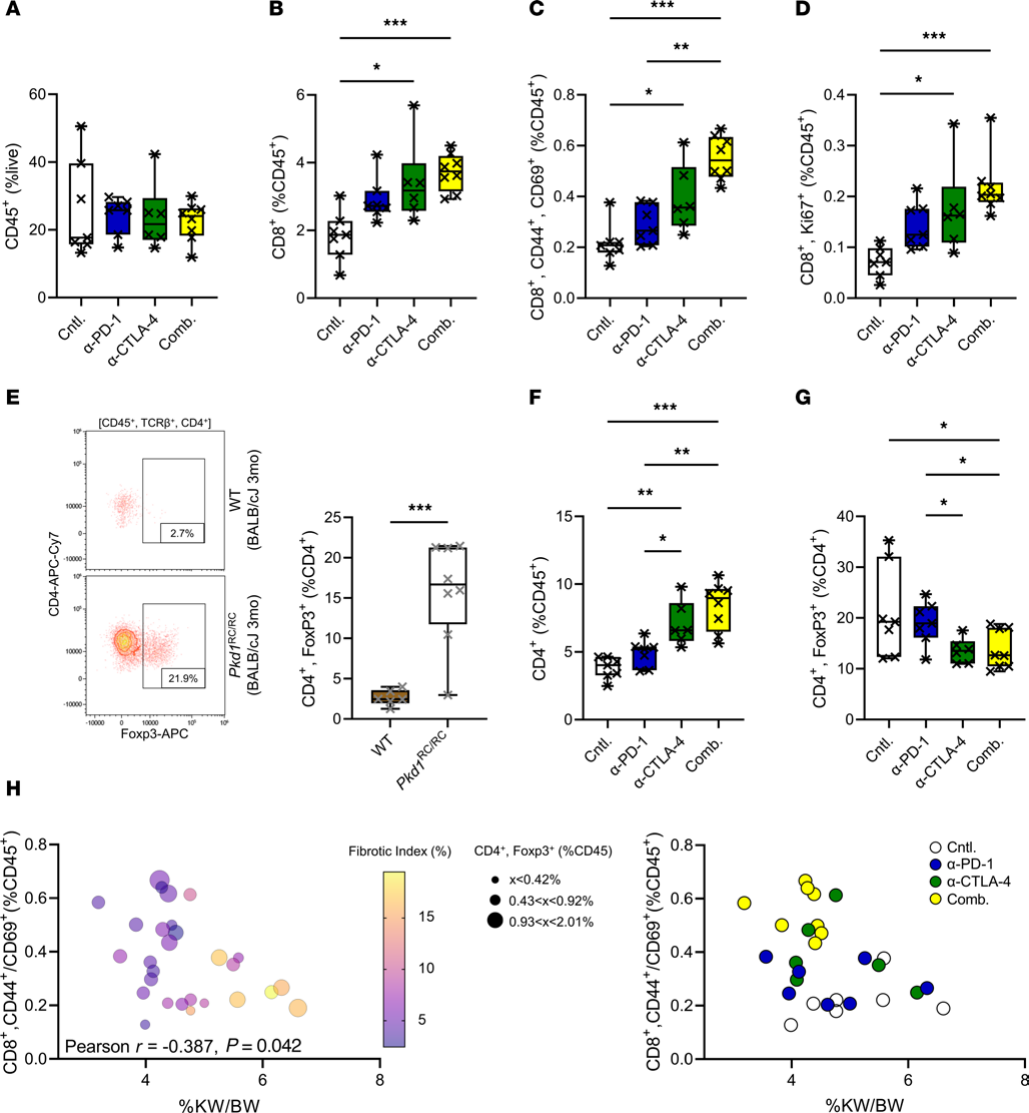
Treatment with α–PD-1 plus α–CTLA-4 results in rebalancing of adaptive immunity within the cystic immune microenvironment. (**A**–**D**, **F**, and **G**) Kidney flow cytometry data from BALB/cJ *Pkd1*^RC/RC^ mice treated with α–PD-1 (blue), α–CTLA-4 (green), or combination (Comb.; yellow); control, white. (**A**) Numbers of immune cells (CD45^+^; % live), (**B**) CD8^+^ T cells (% CD45^+^), and (**C** and **D**) CD8^+^ T cells expressing CD44 and CD69 (**C**) or Ki67 (**D**) (% CD45^+^). α–PD-1 or α–CTLA-4 increased CD8^+^ T cell numbers, activation, and proliferation, which was further amplified by Comb. (**E**) Analysis of Treg numbers in 3-month-old BALB/cJ *Pkd1*^RC/RC^ mice (white) versus WT (brown) shows a significant increase in ADPKD mice. Left: Representative flow diagrams. Right: Quantification. (**F**) CD4^+^ T cell numbers (% CD45^+^). (**G**) CD4^+^ Treg numbers (FoxP3^+^; % CD4^+^). CD4^+^ T cell numbers increased with monotherapy, with additive effects in Comb. Treg numbers decreased with α–CTLA-4 versus control. (**H**) Correlation analyses of CD8^+^ T cell activation and %KW/BW. Left: Data points of individual animals color-coded by severity of fibrotic burden and sized by number of Tregs. Mildest PKD (low %KW/BW, small fibrotic index) correlated with high numbers of activated CD8^+^ T cells plus low numbers of Tregs. Right: Same correlation plot with data points color-coded by treatment (Supplemental Table 5). (**A**–**G**) Box plot (25th to 75th percentile and median) with whiskers of 10th and 90th percentiles; single data points are shown. (**A**–**D**, **F**, and **G**) Kruskal-Wallis 1-way ANOVA with multiple-comparison follow-up by controlling for false discovery rate (Benjamini, Krieger, Yekutieli). (**E**) Nonparametric Mann-Whitney test. (**H**) Pearson’s correlation using 2-tailed multivariate analyses. *P < 0.05, **P < 0.01, ***P < 0.001. (**A**–**D** and **F**–**H**) *N* = 7–8 mice per group. (**E**) *N* = 6–8 mice per genotype. Nonsignificant pairwise comparisons are not shown.

To better understand why monotherapy with either anti–PD-1 or anti–CTLA-4 did not alleviate PKD severity in our model, but combination therapy did, we performed flow cytometry on kidneys at the end of the study to evaluate kidney T cell numbers and activation status as defined by coexpression of CD44 and CD69 (10). In all treatment arms, we did not see a difference in overall kidney immune cell numbers (CD45^+^) compared with control, indicating that these treatments did not globally alter immune cell infiltration into or proliferation within the kidney (Figure 5A). Either anti–PD-1 or anti–CTLA-4 resulted in an increase in kidney CD8^+^ T cell numbers; importantly, there appeared to be an additive effect in animals that received the combination treatment (Figure 5B). When evaluating CD8^+^ T cell activation status, we found that very few CD8^+^ T cells expressed both CD44 and CD69 in the untreated *Pkd1*^RC/RC^ mice (~12% of CD8^+^ T cells). This low frequency of effector CD8^+^ T cells may explain why CD8^+^ T cell loss worsens PKD, as we previously published (10). Either anti–PD-1 or anti–CTLA-4 treatment resulted in a moderate increase of CD8^+^, CD44^+^CD69^+^ T cells in the kidney compared with control, ~32% and ~80% increase, respectively. Again, dual treatment with anti–PD-1 and anti–CTLA-4 increased the numbers of activated CD8^+^ T cells by ~154% compared with control, which is an additional 1-fold increase above the levels achieved by monotherapy (Figure 5C). Similarly, we found a 3-fold increase in proliferating kidney CD8^+^ T cells (Ki67^+^) in animals receiving dual therapy compared with controls, while this increase was only 2-fold in the monotherapy arms (Figure 5D). Together, these data show that although there was no impact on PKD phenotype, either anti–PD-1 or anti–CTLA-4 monotherapy did result in an increase in number and activation status of CD8^+^ T cells. The fact that PKD progression was only slowed in animals receiving combination therapy suggests that there may be a threshold level of CD8^+^ T cell number/function needed to halt cyst growth.

In cancer, it is well established that Tregs suppress anticancer immunity via a multitude of mechanisms (44). Interestingly, Tregs express CTLA-4 as part of their function to maintain immune tolerance. Correlatively, recent cancer studies have shown that treatment with anti–CTLA-4 results in a reduction of intratumoral Tregs, representing a noncanonical mechanism that may contribute to therapeutic efficacy (45, 46). The relevance of Tregs to PKD progression has not been studied, but we recently found an enrichment of this population in PKD kidneys of an adult-onset PKD model caused by inducible kidney-specific *Ift88* loss and in C57BL/6J *Pkd1*^RC/RC^ mice versus controls (18, 19). We confirmed that kidney Treg numbers were significantly increased in BALB/cJ *Pkd1*^RC/RC^ mice at 3 months of age compared with age-, strain-, sex-matched WT mice, the 3-month time point representing the endpoint of our combination therapy study (Figure 5E). Based on these findings, we analyzed the whole CD4^+^ T cell population and the CD4^+^ Treg subpopulation in our anti–PD-1/anti–CTLA-4 study. Kidney CD4^+^ T cell numbers increased as percent CD45^+^, similarly to CD8^+^ T cells, and showed an analogous increase in activation and proliferation status upon combination treatment (Figure 5F and Supplemental Figure 3A). Interestingly, at baseline, the number of CD4^+^ T cells expressing both CD44 and CD69 was much higher compared with CD8^+^ T cells (~47% vs. ~12%, Supplemental Figure 3A vs. Figure 5C). Focusing on Tregs as percent of CD4^+^ T cells, we found a 54% decrease in kidney Treg numbers in animals treated with anti–CTLA-4 or the combination compared with control, paralleling the cancer findings, and suggesting that this effect may have contributed to the efficacy of the combination treatment (Figure 5G). Notably, their activation and proliferation status was not altered by either the single or dual immune checkpoint blockade therapy (Supplemental Figure 3B).

To better understand the relevance of our observed adaptive immune cell changes to PKD severity, we correlated these flow cytometry parameters to %KW/BW, cystic index, and fibrotic index. Intriguingly, the number of CD8^+^ T cells that were activated, as characterized by CD44^+^CD69^+^, was significantly inversely correlated with %KW/BW, cystic index, and fibrotic index (Figure 5H and Supplemental Table 5). Neither CD8^+^ nor CD4^+^ T cell numbers, proliferating CD8^+^ T cells (Ki67^+^), nor Tregs alone significantly correlated with these parameters (Supplemental Table 5). Combining the activation status of CD8^+^ T cells with the proliferative status modestly enhanced the correlation, but the strongest correlation with %KW/BW was achieved when accounting for CD8^+^ T cell activation/proliferation status and Treg numbers, with CD8^+^ activation/proliferation status as a positive variable and Treg number as a negative variable (Supplemental Table 5). Interestingly, consideration of CD4^+^ activation or proliferation status did not improve the correlations (Supplemental Table 5). Together, these data suggest that the observed therapeutic efficacy with dual ICIs is likely to be contributed by both reactivation of the CD8^+^ but not the broad CD4^+^ T cell effector compartment and alleviation of immunosuppressive mechanisms conferred by CD4^+^ Tregs, resulting in a net rebalancing of the adaptive immunity within the CME.

### Expression of CTLA-4|CD80/CD86 and PD-1|PD-L1 occurs on distinct populations in ADPKD kidneys.

**Figure 6:**
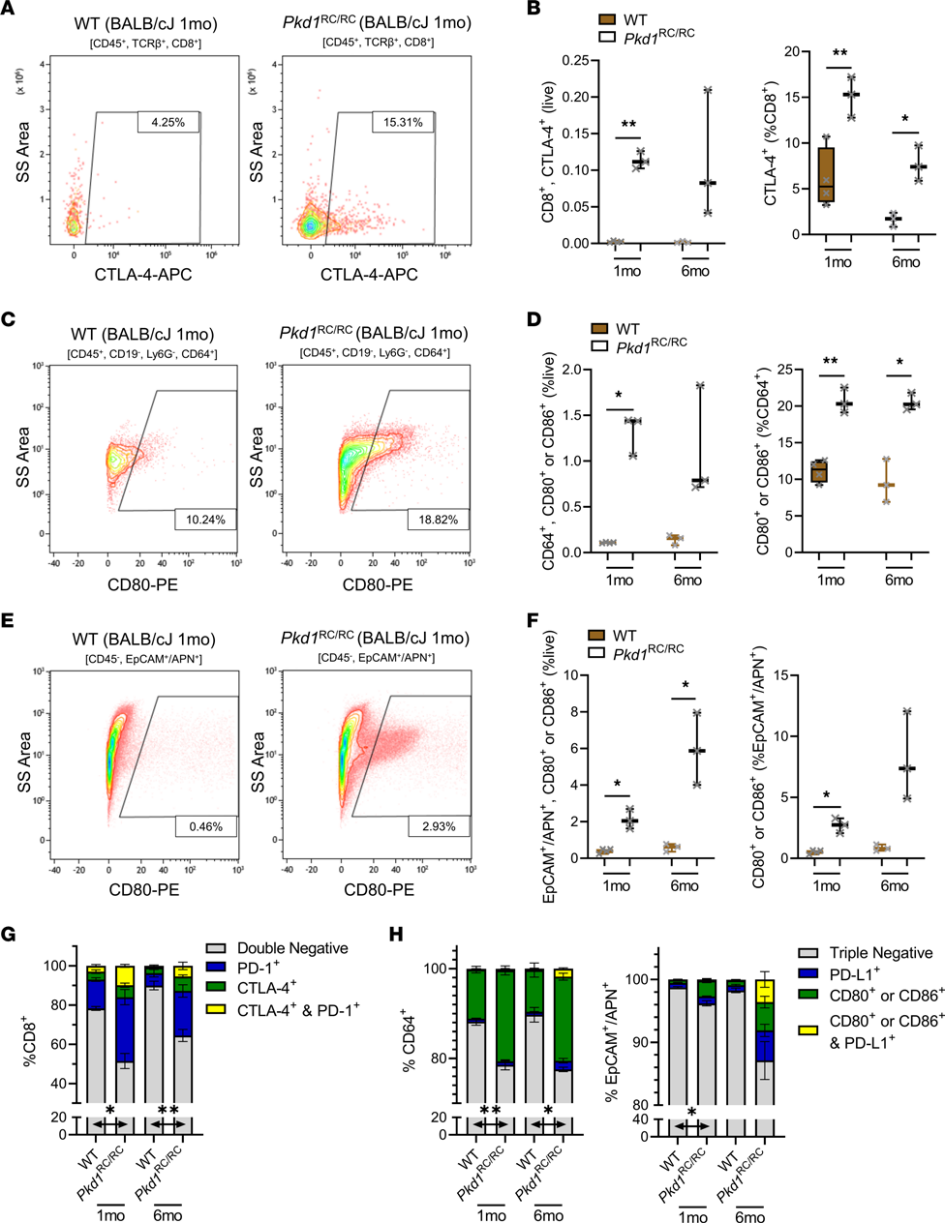
The immune checkpoint proteins CTLA-4|CD80/CD86 are upregulated in ADPKD. Kidneys of BALB/cJ WT and *Pkd1*^RC/RC^ mice were harvested at 1 and 6 months of age and analyzed by flow cytometry for expression of CTLA-4 on CD8^+^ T cells (CD45^+^TCRβ^+^CD8^+^; **A** and **B**), CD80 or CD86 on macrophages (CD45^+^CD19^–^Ly6G^–^CD64^+^; **C** and **D**), and CD80 or CD86 on epithelial cells (CD45^–^EpCAM^+^APN^+^; **E** and **F**). (**A**, **C**, and **E**) Representative flow diagrams of 1-month-old BALB/cJ WT and *Pkd1*^RC/RC^ kidneys. (**B**, **D**, and **F**) Quantification of CTLA-4–positive CD8^+^ T cells (**B**), CD80/CD86–positive CD64^+^ macrophages (**D**), and CD80/CD86–positive epithelial cells (**F**) as percentage live and percentage parent population comparing WT (brown) with *Pkd1*^RC/RC^ (white). CTLA-4 or CD80/CD86 expression is significantly upregulated on the respective cell type in *Pkd1*^RC/RC^ versus WT. (**G**) Analysis of CD8^+^ T cells that express PD-1 (blue), CTLA-4 (green), PD-1 and CTLA-4 (yellow), or no immune checkpoint receptor (gray). (**H**) Analysis of macrophages/epithelial cells that express PD-L1 (blue), CD80/CD86 (green), PD-L1 and CD80/CD86 (yellow), or no immune checkpoint ligand (gray). Few cells coexpress both immune checkpoint proteins on the same cell, suggesting that treatment with antibodies against each immune checkpoint targets different cells. Supplemental Figure 4 contains quantification of CTLA-4 expression on CD4^+^ T cells and individual CD80 or CD86 expression on macrophages/epithelial cells. (**B**, **D**, and **F**) Box plot (25th to 75th percentile and median) with whiskers of 10th and 90th percentiles; single data points are depicted. (**B**, **D**, and **F**–**H**) Brown-Forsythe 1-way ANOVA with multiple-comparison follow-up by controlling for false discovery rate (Benjamini, Krieger, Yekutieli). Nonsignificant pairwise comparisons are not shown. (**G** and **H**) Comparisons are limited to cells expressing neither immune checkpoint protein. *P < 0.05, **P < 0.01. *N* = 4 BALB/cJ WT; *N* = 3 BALB/cJ *Pkd1*^RC/RC^. Data points are male and female.

Our recently published scRNA-Seq data showed that *Ctla4* is expressed in kidney T cells of mice lacking kidney *Ift88* and presenting with slowly progressive PKD (18). Given our above results showing that dual immune checkpoint blockade slows ADPKD1 progression in the BALB/cJ *Pkd1*^RC/RC^ mouse model, reactivates kidney CD8^+^ T cells and CD4^+^ T cells, and lowers kidney CD4^+^ Treg numbers, we undertook studies investigating the expression of CTLA-4 and its ligands within this model. We performed kidney flow cytometry on BALB/cJ WT and *Pkd1*^RC/RC^ mice at 1 and 6 months of age and assayed expression of CTLA-4 on T cell subsets and CD80/CD86 on macrophages or epithelial cells. CTLA-4 expression was significantly increased on both CD8^+^ and CD4^+^ T cells, including CD4^+^ Tregs (marked by CD25; ref. 47), in kidneys of *Pkd1*^RC/RC^ mice compared with WT, linking the effect of single anti–CTLA-4 and dual immune checkpoint blockade to increased CD4^+^ and CD8^+^ T cell numbers, proliferation, and activation, as well as decreased Treg numbers (Figure 6, A and B, and Supplemental Figure 4, A and C). Similarly, we found significantly increased expression of CD80 and CD86 on kidney macrophages and epithelial cells in *Pkd1*^RC/RC^ versus WT mice (Figure 6, C–F, and Supplemental Figure 4, D–I). Intriguingly, our analyses revealed that PD-1 and CTLA-4 were predominantly not expressed on the same populations of CD8^+^ or CD4^+^ T cells; similarly, PD-L1 and CD80/CD86 on macrophages or epithelial cells were not predominantly coexpressed on the same cell (Figure 6, G and H, and Supplemental Figure 4B [compare blue or green vs. yellow]). Further, the population of each cell type that expressed neither immune checkpoint receptor/ligand was significantly decreased in *Pkd1*^RC/RC^ versus WT mice (Figure 6, G and H, and Supplemental Figure 4B [gray]). Together, this differential expression pattern of the 2 immune checkpoint pathway proteins may explain why we only observed therapeutic efficacy of slowing PKD progression with the dual immune checkpoint blockade — treatment with anti–PD-1 plus anti–CTLA-4 allowed targeting of a larger number of total T cells.

**Figure 7:**
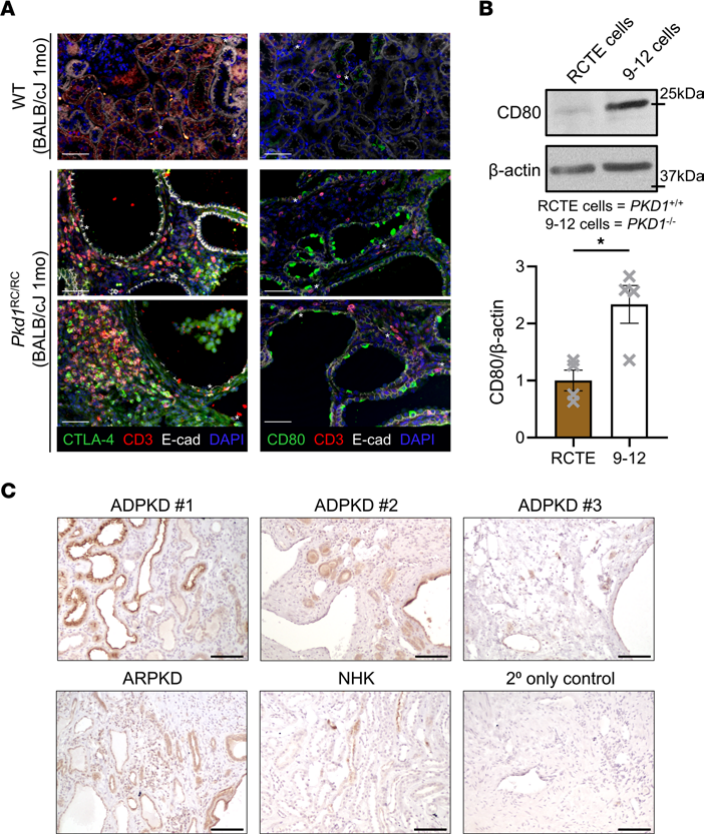
CTLA-4|CD80/CD86 immune checkpoint protein expression is increased at cystic lesions of *Pkd1*^RC/RC^ kidneys and in human ADPKD samples. (**A**) Immunofluorescence (IF) labeling of CTLA-4 (left) and CD80 (right) in BALB/cJ WT and *Pkd1*^RC/RC^ kidneys. Merged IF image is shown; Supplemental Figures 5 and 6 contain single-channel images. CTLA-4 or CD80 (green), T cells/CD3 (red), epithelial cells/E-cadherin (white), nuclei/DAPI (blue). In *Pkd1*^RC/RC^ but not WT kidneys, T cells stain positive for CTLA-4 and are in proximity to cystic lesions. *Pkd1*^RC/RC^ sections: T cells positive for CTLA-4 differ from ones positive for PD-1; compared with Figure 2A, serial sections were stained. Cystic epithelial cells stain strongly positive for CD80 in *Pkd1*^RC/RC^ but not WT kidneys. Asterisks in WT: T cells; asterisks in *Pkd1*^RC/RC^: CTLA-4–positive T cells in contact with epithelial cells, CD80-positive interstitial or epithelial cells in contact with T cells. *Pkd1*^RC/RC^: 2 separate animals are shown. Scale bars: 50 μm. (**B**) CD80 expression in immortalized human RCTE cells (*PKD1^+/+^*) versus 9-12 cells (*PKD1^–/–^*). Band size (kDa) of kidney CD80 correlates with prior publications (72). 9-12 cells have increased levels of CD80 compared with control. Top: Representative Western blot image. Bottom: Quantification of Western blots from 4 independent samples. (**C**) IHC staining for CD80 in end-stage kidney tissue of 3 ADPKD patients, an ARPKD patient, and a normal human kidney (NHK). 2° only control: kidney tissue slide of an ADPKD patient stained without addition of primary antibody. Scale bars: 100 μm. The tubular/cystic epithelium in ADPKD and ARPKD samples shows increased expression of CD80 compared with CD80 expression in tubules of NHK. Data are presented as mean ± SEM (**B**); single data points are depicted. An unpaired 2-tailed *t* test was performed (**B**). *P < 0.05.

We also evaluated the expression of CTLA-4 and CD80 at cystic lesions and in the setting of human disease by immunostaining; technical difficulties with detection prevented us from evaluating CD86 expression. In 1-month-old BALB/cJ *Pkd1*^RC/RC^ kidneys, we found ample T cells (CD3^+^) expressing CTLA-4 at cystic lesions, some of which were in direct contact with the cystic epithelium (Figure 7A and Supplemental Figure 5). Notably, T cells positive for either CTLA-4 or PD-1 appeared to be distinct populations, coinciding with our flow cytometry findings (*Pkd1*^RC/RC^ sections: comparing Figure 2A vs. Figure 7A, staining of serial sections). T cells in WT kidneys did not stain positive for CTLA-4 (Figure 7A and Supplemental Figure 5). Similarly, WT kidneys showed only weak staining for CD80, while *Pkd1*^RC/RC^ kidneys had very pronounced cystic epithelial staining, with some interstitial cells that surrounded the cysts staining positive as well (Figure 7A and Supplemental Figure 6). We performed Western blotting for CD80 on lysates obtained from immortalized *PKD1^+/+^* cells (RCTE) and *PKD1^–/–^* cells (9-12 cells). We found a significant increase of CD80 in 9-12 compared with RCTE cells, although this increase was less pronounced than that of PD-L1 (Figure 7B and Figure 2B). Similarly, we found clear epithelial CD80 expression in end-stage kidney sections from patients with PKD by IHC (ADPKD and ARPKD), which differed in intensity from that in NHK tissue sections (Figure 7C). Together, these data suggest that the CTLA-4|CD80/CD86 immune checkpoint is dysregulated similarly to the PD-1|PD-L1 checkpoint in murine and human ADPKD and that both checkpoints likely impact the function of distinct cell subpopulations.

## Discussion

We have shown that the number of kidney CD8^+^ T cells increases with PKD severity and that depletion of CD8^+^ T cells worsens disease outcome (10). This suggests that this population of adaptive immune cells functions in halting cyst growth. It is known that CD8^+^ T cells lose functionality through engagement of immune checkpoints. Here, we explored the contribution of 2 immune checkpoints, PD-1|PD-L1 and CTLA-4|CD80/CD86, to kidney cyst growth using different orthologous ADPKD1 models with varying rates of PKD progression. Using flow cytometry of kidney single-cell suspensions, we found significant upregulation of PD-1/CTLA-4 on kidney CD8^+^ T cells in ADPKD1 mice compared with control (Figures 1 and 6). Importantly, T cells expressing either of the immune checkpoint receptors localized to cystic lesions in mouse ADPKD1 kidneys, while T cells in WT kidneys were largely negative for either PD-1 or CTLA-4 (Figures 2 and 7). Further, we found increased expression of PD-L1 or CD80/CD86 on kidney epithelial cells and macrophages using flow cytometry and immunofluorescence imaging (Figures 1, 2, 6, and 7 and Supplemental Figure 4). Upregulated expression of PD-L1/CD80 was also verified in ADPKD cell lines and patient kidney samples versus controls (Figures 2 and 7). This suggests that CD8^+^ T cells may be inhibited by these pathways within the CME and likely lose their proposed anti-cystogenic function. These findings are consistent with our recently published scRNA-Seq data that suggest a direct communication between kidney T cells and kidney epithelial cell/mononuclear phagocytes using ligand/receptor analyses as well as show an enrichment of CD8^+^ T cells that express the PD-1 transcript (18). Following the cancer paradigm, upregulation of PD-L1 on kidney macrophages and epithelial cells implies an active commitment to drive immunosuppression, which would allow for the cystic epithelium to escape immune-mediated killing. However, PD-L1 upregulation could also be involved in protecting the epithelium from immune-mediated injury, as seen in the case of lupus nephritis, or it could simply be a concomitant of epithelial cell remodeling.

We verified the induction of the PD-1|PD-L1 immune checkpoint pathway in *Pkd1*^RC/RC^ mice inbred into 3 different strains (C57BL/6J, BALB/cJ, and 129S6/SvEvTac; Figure 1). This demonstrates that the immune checkpoint is activated with progressive PKD independent of the rate of cyst growth — as BALB/cJ and 129S6/SvEvTac *Pkd1*^RC/RC^ mice present with more rapidly progressive adult-onset PKD than C57BL/6J *Pkd1*^RC/RC^ mice. Further, it provides evidence that inhibition of kidney CD8^+^ T cell function is characteristic of PKD independent of the immune cell composition within the kidney, which differs significantly in regard to immune cell types and numbers in different mouse strains as has been shown by us in the case of kidney CD4^+^ to CD8^+^ T cell ratio (10, 48, 49). Hence, our data support that upregulation of immune checkpoint proteins within the CME is a robust finding independent of the rate of PKD progression or mouse strain and suggest that immunosuppression is a critical feature of the PKD kidney. Several lines of evidence have suggested a similar phenotype of the PKD CME. For example, ample data demonstrate that kidney macrophages in PKD models have an M2-like phenotype that mimics that of TAMs (7, 9). This includes findings that indicate that PKD macrophages express characteristic markers such as CD206, as well as upregulate the expression of ARG1, IL-10, CCR2, and CSF1R (50–53). In line with these findings, depletion of macrophages as well as inhibition of CCR2 or CSF1R has been shown to slow cyst growth in inducible early-onset or inducible late-onset PKD models (7, 9, 51, 52). Beyond macrophages, our previous publications using inducible kidney-specific *Ift88-*knockout mice and C57BL/6J *Pkd1*^RC/RC^ mice and our present findings using BALB/cJ *Pkd1*^RC/RC^ mice (Figure 5E) highlight an enrichment of Tregs in PKD kidneys (18, 19). Tregs, just like TAMs, are known drivers of immunosuppression in cancer (44). Last, we recently reported increased expression of immunosuppressive IDO1 in the setting of ADPKD, inhibition of which resulted in slowed disease progression (19).

Surprisingly, inhibiting PD-1|PD-L1 both genetically and pharmacologically had no impact on disease severity (Figure 3). We verified these negative findings extensively focusing on both the receptor (PD-1) and the ligand (PD-L1) and testing the importance of the pathway to PKD pathogenesis in early-onset (*Pkd1*^RC/–^) as well as adult-onset (*Pkd1*^RC/RC^) ADPKD models. These results led us to conclude that redundancy in immune checkpoint activation may be critical in the setting of ADPKD. Indeed, using a combination therapy approach of anti–PD-1 and anti–CTLA-4 in BALB/cJ *Pkd1*^RC/RC^ mice slowed PKD progression compared with either monotherapy arm or control (Figure 4). These findings were in line with our data that the CTLA-4|CD80/CD86 immune checkpoint proteins are increased in ADPKD1 compared with control (Figures 6 and 7) and current clinical findings from the cancer literature that show increased efficacy of combination therapy with FDA-approved anti–PD-1 plus anti–CTLA-4 versus monotherapy alone (18, 20, 35, 42, 43).

Further, in line with cancer findings, kidney flow cytometry analyses of animals treated with both ICIs showed a clear reactivation of the effector T cell compartment, with increased CD8^+^ T cell numbers, activation, and proliferation. In addition, we further observed a decline in the immunosuppressive Treg population associated with anti–CTLA-4 treatment (Figure 5). Correlation analyses with PKD phenotypes suggest that both increased CD8^+^ T cell activation and decreased Treg numbers yielded the largest impact on slowing PKD progression. Notably, we did observe increased activation/proliferation of CD4^+^ T cells with ICI therapy; however, inclusion of the changes to the CD4^+^ T cell compartment, beyond Treg number, did not strengthen our correlation analyses, suggesting that the phenotypic effects on PKD outcome are largely driven by CD8^+^ T cells and CD4^+^ Tregs (Supplemental Table 5). Seeing a therapeutic effect in slowing kidney cyst growth with the combined immune checkpoint blockade and an associated remodeling of adaptive immunity not only provides functional evidence that immune checkpoint activation is a pathogenic driver of PKD, but also reemphasizes the role of adaptive immune cells, specifically CD8^+^ T cells, in modulating PKD progression. It should be noted that the therapeutic effect of dual immune checkpoint blockade, while significant, was only modest, signifying that immunosuppression is only one of many pathways driving kidney cyst growth and accentuating the potential to combine therapeutic approaches that target both epithelial cells and cells within the CME.

Some caution is warranted when contemplating ICIs for PKD. In patients with cancer ICI use has been associated with a variety of autoimmune adverse events involving the skin, the gastrointestinal tract, the endocrine system, and, to a lesser extent, the kidney. Approximately 2%–3% of patients with cancer receiving ICI monotherapy and about 5% receiving ICI combination therapy present with acute kidney injury (AKI) (54, 55). Speculatively, ICIs break self-tolerance and trigger an autoimmune response against self-antigens, which leads to immune-mediated injury in the kidney. While AKI can be managed clinically, this side effect cannot be ignored in the case of PKD patients, as kidney injury has been proposed to accelerate cyst growth in inducible PKD models (56, 57). Hence, long-term use of ICIs, as would be needed for the treatment of ADPKD, is likely not translatable. However, several cancer studies suggest a durable response and progression-free survival after discontinuation of mono- or combination ICI therapy for 6–12 months depending on the type of cancer (58–60). The underlying mechanisms associated with this treatment-free survival remain unclear but likely correspond to a permanent remodeling of the tumor microenvironment associated with innate and adaptive immune memory. Consequently, an altered dosing regimen that does not require continual long-term treatment but instead initial challenges and rechallenges with ICIs may be feasible to translate to the ADPKD clinic. However, the efficacy or side effects of such protocols were not tested in our studies and are part of future work alongside investigations focused on alternative pathways that induce immunosuppression and CD8^+^ T cell exhaustion, which may be targetable alone or in combination with ICIs or other epithelium-centric drugs such as tolvaptan (JYNARQUE), currently the only FDA-approved therapy for ADPKD.

Our study has limitations. For one, we heavily focused on the *Pkd1* p.R3277C model. While we used mice spanning the full spectrum of ADPKD severity seen in patients, from early-onset (C57BL/6J *Pkd1*^RC/–^) to late-onset (C57BL/6J *Pkd1*^RC/RC^) PKD, the underlying disease driver was always a germline modification impacting polycystin-1 dosage. It is plausible that the therapeutic outcome of intervening with PD-1|PD-L1 signaling may differ when using other models of PKD such as germline *Pkd2* or ciliopathy models, as well as inducible kidney-specific *Pkd1* or *Pkd2* knockout models. It is important, though, to keep the relevance of the model to human disease in mind, which was our priority when choosing the *Pkd1* p.R3277C model for these studies. Not only does the CME composition differ between rapidly progressive and slowly progressive models of PKD, as we have previously highlighted, it is also conceivable that the activation status/functionality of kidney immune cells varies significantly between germline and inducible PKD models (10). In PKD models driven by germline mutations, kidney immune cells become conditioned to cyst-induced kidney remodeling from early embryogenesis on, while in inducible models the kidney immune cells remain naive to cyst growth–associated changes prior to PKD induction, changing the time scale for immune responses drastically. Our study was also limited in the cell types analyzed that express PD-1|PD-L1 or CTLA-4|CD80/CD86. We focused our analyses predominantly on the most studied and best characterized cell types, CD8^+^ T cells for PD-1/CTLA-4 and epithelial cells/macrophages for PD-L1/CD80/CD86. However, the expression spectrum of PD-1/CTLA-4 on adaptive immune cells goes far beyond CD8^+^ T cells. Notably, we also found increased expression of PD-1 and CTLA-4 on CD4^+^ T cells when comparing ADPKD1 mice to WT, but the functional complexity of this T cell population made it difficult to draw firm conclusions with regard to the functional relevance of CD4^+^ T cell immune checkpoint receptor expression (Supplemental Figure 4 and Supplemental Table 6). Beyond T cells, the receptors are also expressed on B cells and on natural killer T cells as well as on antigen-presenting cells (APCs) and tumor cells, where their expression, in part, has been associated with ICI therapy resistance (61). PD-L1 is also expressed on APCs beyond macrophages, such as dendritic cells, as well as effector T cells, where its functional relevance has been less well defined. We also did not evaluate the expression of CTLA-4|CD80/CD86 in the setting of PD-1|PD-L1 inhibition in our orthologous models. As mentioned, it is possible that anti–PD-1 monotherapy showed no therapeutic efficacy due to activation of other immune checkpoints upon PD-1|PD-L1 blockade, e.g., upregulation of CTLA-4|CD80/CD86. Therefore, more detailed investigations of cell type–specific expression of these immune checkpoint proteins and others, such as TIM-3 and LAG-3, and their relevance to PKD are still needed. Further, it would be important to prove that beyond activation and proliferation, CD8^+^ T cells also gained cytolytic activity and production of effector cytokines upon immune checkpoint blockade. Similarly, it would be interesting to identify changes in effector CD4^+^ T cell subpopulations such as Th1 or Th17 cells as well as innate immune cell populations upon immune checkpoint blockade, neither of which was part of our studies. Last, we did not delineate the mechanisms underlying increased/persistent immune checkpoint receptor/ligand expression in ADPKD. It would be suggestive if increased PD-1/CTLA-4 expression on T cells is a response to chronic antigen stimulation, driven by cystic epithelial cell–produced neoantigens due to genomic instability or injury-induced damage-associated molecular patterns within the CME (62). Similarly, multiple pathways implicated in PKD (e.g., IFN-γ/JAK-STAT, TNF-α, NF-κB, PI3K, and HIF-1) drive PD-L1 and CD80/CD86 expression on tumor cells (10, 63–66). Hence, while our study provides critical foundational insight that immunosuppression (i.e., immune checkpoint activation) is a characteristic of the cystic kidney that functions in modulating disease severity, much more research is needed in understanding the underlying mechanism driving this phenomenon and targeting it therapeutically. Notably, a recent publication suggests that absolute numbers of peripheral blood CD4^+^ Tregs, PD-1–positive CD8^+^ T cells, and CTLA-4–positive Tregs are increased in patients with ADPKD compared with healthy controls, paralleling our findings and providing additional relevance of our study to the human disease (67).

## Methods

Full methods and antibody information are available in Supplemental Methods.

### Murine study details, experimental models, and genetic crosses

Fully inbred, homozygous C57BL/6J, 129S6/SvEvTac, and BALB/cJ *Pkd1*^RC/RC^ (Pkd1^tm1.1Pcha^) mice and inbred, heterozygous C57BL/6J *Pkd1^del2/+^* (Pkd1^tm1Shh^) mice were obtained from Peter C. Harris (Mayo Clinic, Rochester, Minnesota, USA) (10, 33, 34, 36, 68). Fully inbred, homozygous C57BL/6J and BALB/cJ *Cd274*-knockout (Cd274^tm1Lpc^) mice were obtained from Haidong Dong (Mayo Clinic) (69).

C57BL/6J *Pkd1*^RC/–^ mice were obtained by crossing of C57BL/6J *Pkd1*^RC/RC^ with C57BL/6J *Pkd1^del2/+^* mice. C57BL/6J or BALB/cJ *Pkd1*^RC/RC^ *Cd274^+/+^* and *Pkd1*^RC/RC^ *Cd274^–/–^* animals were obtained by crossing of strain-matched F_1_ *Pkd1*^RC/RC^ *Cd274^+/–^* pups. Housing and genotyping information is outlined in Supplemental Methods.

For all studies, males and females were used. Statistical analyses revealed no difference between males and females regarding evaluated PKD phenotypes; hence, both sexes were combined for all analyses.

### Human samples

Deidentified human ADPKD/autosomal recessive PKD (ARPKD) and NHK FFPE sections were obtained through a material transfer agreement with the Kansas PKD Research and Translational Core Center (P30DK106912) at the University of Kansas Medical Center (Kansas City, Kansas, USA). The PKD samples were end-stage kidney samples collected after transplantation.

### Cell culture

All cell lines have been previously described (70): renal cortical tubular epithelial (RCTE) cells, *PKD1^+/+^*, and 9-12 cells, *PKD1^–/–^*. Cells were grown in DMEM/Ham’s F-12, 50:50, mixed with l-glutamine and 15 nM HEPES (DMEM/F12; Corning, MT10092CV) supplemented with 10% FBS (Sigma-Aldrich, MFCD00132239) and 1% penicillin-streptomycin (Corning, MT30002CI) for fewer than 10 passages.

### Immunoblotting

RCTE and 9-12 cells were grown until about 70%–80% confluence, and protein was isolated. A total of 30 μg of protein was analyzed on a 10% SDS polyacrylamide gel, transferred to a PVDF membrane, and incubated with primary antibody overnight. The membranes were exposed to ECL reagent (PerkinElmer, NEL104001EA) and developed using an x-ray film developer. Band density of each blot was quantified using ImageJ software (NIH). Antibodies are in Supplemental Methods.

### Immunohistochemistry

Human kidney ADPKD, ARPKD, and NHK sections were stained using the VectaStain Elite ABC Universal Plus kit (Vector Laboratories, PK-8200). Antibodies are in Supplemental Methods.

### Immunofluorescence

Tissues were prepared for immunofluorescence labeling as previously described (10, 19, 33). Antibodies are in Supplemental Methods. The slides were visualized using a Keyence BZ-X710.

### Histomorphometric and kidney function analyses

Cystic index, cyst size, and cyst number were analyzed as previously published (10). Fibrotic area was analyzed from Picrosirius red–stained kidney sections visualized using an Olympus BX41 microscope with a linear polarizer as previously published (10).

BUN levels were analyzed following the manufacturer’s protocol (QuantiChrom Urea Assay Kit; BioAssay Systems, 501079333). Samples were analyzed in duplicates.

### Immunodepletion experiments

#### Anti–PD-1 studies.

Four-month-old 129S6/SvEvTac *Pkd1*^RC/RC^ mice were treated twice a week for 8 weeks by i.p. injection with 10 mg/kg anti–PD-1 blocking antibody (clone RMP1-14; Bio X Cell, BP0146) or 10 mg/kg IgG2a control (clone 2A3; Bio X Cell, BP0089). C57BL/6J *Pkd1*^RC/–^ mice were treated every other day by i.p. injection with 10 mg/kg anti–PD-1 blocking antibody or 10 mg/kg IgG2a control starting at P8 until P20.

#### Anti–PD-1/anti–CTLA-4 study.

One-month-old BALB/cJ *Pkd1*^RC/RC^ mice were treated twice a week for 8 weeks by i.p. injection with 10 mg/kg anti–PD-1 blocking antibody, 10 mg/kg anti–CTLA-4 blocking antibody (clone 9D9; Bio X Cell, BP0164), the combination of anti–PD-1 and anti–CTLA-4, or control IgG (10 mg/kg IgG2a and 10 mg/kg IgG2b control [clone MPC-11; Bio X Cell, BP0086]). Animals with single blockade of PD-1 or CTLA-4 also received the respective other control antibody.

### Single-cell suspension and flow cytometry

Single-cell suspensions of the dissected kidneys were prepared as previously described (10, 19). In short, tissue was mechanically dissociated and digested in DMEM/F12 media (Corning, MT15090CV) containing Liberase TL (2 mg/mL; Sigma-Aldrich, 05401020001) and DNase I (20,000 U/mL; Sigma-Aldrich, D5025) for 30 minutes at 37°C, then passed through a 100 μm and 70 μm filter, as well as cleared of red blood cells using red blood cell lysis buffer (0.015 M NH_4_Cl, 10 mM KHCO_3_, 0.1 mM Na_2_EDTA, pH 7.2).

Staining of single-cell suspension and flow cytometry protocol were described previously (10). Each kidney cell suspension was split in half and stained with 2 different panels (T cell panel, epithelial/macrophage panel; for antibodies, see Supplemental Methods). The single-cell suspension was blocked in anti–mouse CD16/CD32 (clone 93; eBioscience, 14-0161-86) for 15 minutes, followed by viability staining (LIVE/DEAD Fixable Aqua Dead Cell Stain Kit; Invitrogen, L34966) for 15 minutes and conjugated surface antibody staining for 30 minutes. Intracellular markers were stained for 2 hours with the Foxp3/Transcription Factor Staining Buffer Set (eBioscience, 00-5523-00).

After staining, cells were run on a Gallios Flow Cytometer Machine (Beckman Coulter) and analyzed using Kaluza Analysis v2.1 software (Beckman Coulter). The analysis workflow and gating strategies are outlined in Supplemental Methods.

### Statistics

All analyses were performed using JMP Pro 16.1 (SAS) or Prism 9.2.0 (GraphPad Software). Data are presented as mean ± SEM or box plot (25th to 75th percentile and median) with whiskers of 10th and 90th percentiles; single data points are depicted. Pairwise comparisons were performed using a Mann-Whitney test, and multigroup comparisons were performed using Kruskal-Wallis test or Brown-Forsythe test (if *N* < 4) with multiple-comparison follow-up by controlling for false discovery rate (Benjamini, Krieger, Yekutieli). The Pearson’s correlation coefficient was computed using 2-tailed multivariate analyses with a confidence interval of 95%. Significant *P* values are denoted by *P < 0.05, **P < 0.01, ***P < 0.001, and ****P < 0.0001.

### Study approval

All experimental procedures were performed in an Association for Assessment and Accreditation of Laboratory Animal Care International–accredited facility in accordance with the *Guide for the Care and Use of Laboratory Animals* (71) and approved by the University of Colorado Anschutz Medical Campus Institutional Animal Care and Use Committee (protocols 33, 301, 685).

### Data availability

All underlying data generated as part of this study are available from the corresponding author upon request and executed data transfer agreement through the University of Colorado Anschutz Medical Campus Office of Contracts.

## Author contributions

EKK, ETC, RAN, and KH conceived and designed research. EKK, DTN, KHM, CDB, ASL, MLTM, MDB, SBF, and KH performed experiments. EKK, DTN, and KH analyzed data. EKK, DTN, ETC, KAZ, RAN, and KH interpreted results of experiments. EKK and KH prepared figures. EKK and KH drafted the manuscript. EKK, DTN, ASL, MLTM, SBF, BYG, MBC, ETC, KAZ, and RAN edited and revised the manuscript. Authorship order among EKK and DTN, co–first authors, was decided based on overall time/effort commitment and intellectual contribution to the study.

## Supplementary Material

Supplemental data

## Version 1 06/22/2023

Electronic publication
