# Supplementary material for: Immune checkpoint activity regulates polycystic kidney disease progression
Source: JCI Insight. 2023 Jun 22;8(12):e161318. doi: 10.1172/jci.insight.161318 (PMC10371237; doi:10.1172/jci.insight.161318)
Supplement: Supplemental data [file jciinsight-8-161318-s124.pdf]

SUPPLEMENTAL MATERIAL

Immune Checkpoint Signaling Regulates Polycystic Kidney Disease Progression

Emily K. Kleczko[1+]; Dustin T. Nguyen[1+]; Kenneth H. Marsh[1]; Colin D. Bauer[1]; Amy S. Li[1]; Marie-Louise T. Monaghan[1]; Michael D. Berger[1]; Seth B. Furgeson[1]; Berenice Y. Gitomer[1]; Michel B. Chonchol[1,2]; Eric T. Clambey[3]; Kurt A. Zimmerman[4]; Raphael A. Nemenoff[1,2]; Katharina Hopp[1,2*]

[1]Department of Medicine, Division of Renal Diseases and Hypertension, University of Colorado Anschutz Medical Campus, Aurora, CO 80045, USA

[2]Consortium for Fibrosis Research and Translation, University of Colorado Anschutz Medical Campus, Aurora, CO 80045, USA

[3]Department of Anesthesiology, University of Colorado Anschutz Medical Campus, Aurora, CO 80045, USA

[4]Department of Internal Medicine, Division of Nephrology, University of Oklahoma Health Sciences Center, Oklahoma City, OK 73104, USA

[+]Contributed equally to this work.

***Corresponding Author:**

Dr. Katharina Hopp

Division of Renal Diseases and Hypertension

University of Colorado Anschutz Medical Campus

12700 E 19th Avenue

P15-7400A

Mail Stop C281

Aurora, CO 80045

Phone: 303-724-1858

Fax: 303-724-4868

E-mail: katharina.hopp@cuanschutz.edu

SUPPLEMENTAL FULL METHODS

Murine Study Details, Experimental Models, and Genetic Crosses

All study animals were group-housed, separated by sex, with a maximum of five animals per cage, maintained on a standard diet (ENVIGO #2920), and had food plus water freely available. The housing facility is pathogen-free and was kept at a temperature of ~72ºF, humidity of ~37%, and a light cycle of 8pm off, 6am on.

Fully inbred, homozygous C57Bl/6J, 129S6/SvEvTac, and BALB/cJ *Pkd1*$^{RC/RC}$ mice (Pkd1$^{tm1.1Pcha}$) were obtained from Dr. Peter C. Harris, Mayo Clinic, Rochester, MN, USA in 2015 with an approved material transfer agreement (MTA) and were maintained in homozygosity by the principal investigator (PI), Katharina Hopp(1-4). Homozygous *Pkd1*$^{RC/RC}$ mice were outcrossed every 10th generation to strain-matched wildtype mice obtained from *The Jackson Laboratory* (stock number 000664 [C57Bl/6J]; 000651 [BALB/cJ]) or *Taconic Biosciences* (model number 126SVE). Inbred, heterozygous C57Bl/6J *Pkd1*$^{del2/+}$ (Pkd1^{tm1Shh}) mice were obtained under the same MTA mentioned above and maintained in heterozygosity by crossing with C57Bl/6J wildtypes (*The Jackson Laboratory*, stock number 000664)(5).

Fully inbred, homozygous C57Bl/6J and BALB/cJ *Cd274* knockout mice (PD-L1, Cd274^{tm1Lpc}) were obtained in 2017 from Dr. Haidong Dong (Mayo Clinic, Rochester, MN, USA) with the appropriate MTA in place(6). They were maintained in homozygosity and used for all experiments without outcrossing.

C57Bl/6J *Pkd1*$^{RC/-}$ mice were obtained by crossing C57Bl/6J *Pkd1*$^{RC/RC}$ and C57Bl/6J *Pkd1*$^{del2/+}$ mice. C57Bl/6J or BALB/cJ *Pkd1*$^{RC/RC}$;*Cd274*$^{+/+}$ and *Pkd1*$^{RC/RC}$;*Cd274*$^{-/-}$ animals were generated by crossing strain-matched F1 *Pkd1*$^{RC/RC}$;*Cd274*$^{+/-}$ pups obtained from four different *Pkd1*$^{RC/RC}$ and *Cd274*$^{-/-}$ parental crosses. In total, six different F1 crosses generated all F2 experimental animals (C57Bl/6J or BALB/cJ *Pkd1*$^{RC/RC}$;*Cd274*$^{+/+}$ and *Pkd1*$^{RC/RC}$;*Cd274*$^{-/-}$).

The genotypes of all experimental animals were PCR confirmed using DNA extracted from tail clips. The *Pkd1* p.R3277C genotyping protocol was performed as previously published(2). The *Pkd1* del2 genotyping was performed using a wildtype (5'-ACGCTGGGCAAAGGAACATC-3'; 5'- TGGGAACAGAGAGAGACAGTGGTC-3'; 380bp) and mutant (5'- CGACCACCAAGCGAAACATC-3'; 5'-GTCCGACATTGCTCCTGTGC-3'; 475 bp) PCR reaction. Both PCRs were run using the same conditions (94°C 3min; 35x 94°C 30sec, 59°C 30sec, 72°C 40sec;

72°C 10min; 4°C 10min). The *Cd274* genotyping was performed as multiplex PCR (5'-AGAACGGGAGCTGGACCTGCTTGCGTTAG-3'; 5'-ATTGACTTTCAGCGTGATTCGCTTGTAG-3'; 5'-TCTATGGCTTCTGAGGCGGA-3'; wildtype: 250bp, mutant 160bp) using the following PCR conditions: 94°C 3min; 35x 94°C 30sec, 58°C 30sec, 72°C 50sec; 72°C 10min; 4°C 10min.

For all studies, both sexes, males and females, were utilized. Statistical analyses revealed no difference between males and females regarding evaluated PKD phenotypes; hence, both sexes were combined for all analyses. All animals were aged as outlined in the results sections prior to euthanasia and PKD parameter analyses were performed as outlined below. The number of animals per study varied and is indicated in the respective results sections and figure legends.

Human Samples

De-identified human ADPKD/Autosomal Recessive PKD (ARPKD) and normal human kidney (NHK) FFPE sections were obtained through an MTA with the Kansas PKD Research and Translational Core Center (P30 DK106912) at the University of Kansas Medical Center (Kansas City, KS, USA). The PKD samples are end-stage kidney samples which were collected post-transplant.

Cell Culture

All cell lines have been previously described and were originally purchased from ATCC, CRL-2833(7). Briefly, renal cortical tubular epithelial (RCTE) cells are *PKD1*$^{+/+}$ immortalized human renal cortical tubular epithelial cells, and 9-12 cells are *PKD1*$^{-/-}$ immortalized cells derived from human ADPKD cystic epithelium. Cells were grown in Dulbecco's modified Eagle's medium/Ham's F-12 50/50 mixed with L-glutamine and 15nM HEPES (DMEM/F12; *Corning,* #MT10092CV) supplemented with 10% fetal bovine serum (*Sigma-Aldrich,* #MFCD00132239) and 1% penicillin-streptomycin (*Corning,* #MT30002CI). Cells were grown in a humidified incubator with 5% CO_2 at 37°C. Cells were kept in culture for no longer than 6-8 weeks and up to 10 passages at maximum before a new vial was thawed.

Immunoblotting

RCTE and 9-12 cells were grown on tissue-culture treated 10cm plates until ~70-80% confluency, after which cells were harvested and lysed (0.5% Triton X-100, 50 mM β-glycerophosphate [pH 7.2], 0.1 mM Na_3VO_4, 2mM $MgCl_2$, 5 mM EDTA, plus protease inhibitor cocktail [*Sigma-Aldrich,* cOmplete™, #P8340]). Protein concentration was measured using protein assay dye reagent concentrate (*Bio-Rad*, #5000006): Bradford method. A total of 30µg of protein were loaded on a 10% sodium dodecyl-sulfate polyacrylamide gel and transferred onto a polyvinylidene difluoride membrane. Membranes were blocked in 5% bovine serum albumin in Tris-buffered saline with 0.1% Tween 20 (TBST) for 1 hour, incubated overnight at 4°C with primary antibodies, and for 1 hour with secondary antibody at room temperature. For probing of different antibodies, membranes were stripped using Restore Western Blot Stripper Buffer (*Thermo Scientific,* # 21059) according to the manufacturer's instructions. The membranes were exposed to ECL reagent (*Perkin Elmer*, #NEL104001EA) and developed using an X-ray film developer. Band density of each blot was quantified using ImageJ software. Antibodies: rabbit anti-PD-L1 (*Cell Signaling Technology*, #13684; 1:1,000), rabbit anti-CD80 (*Abcam*, #ab254579; 1:1,000), goat anti-rabbit-HRP (*Pierce*, #1858415; 1:5,000), mouse anti-β-actin (*Sigma-Aldrich,* #A5441; 1:10,000), goat anti-mouse-HRP (*Jackson ImmunoResearch Laboratories Inc*, #115-035-003; 1:20,000).

Immunohistochemistry

Human kidney ADPKD, ARPKD and NHK sections were stained following the VectaStain Elite ABC Universal Plus kit (*Vector Laboratories*, #PK-8200). Antigen unmasking was performed with slides submerged in Universal HIER antigen retrieval reagent (PD-L1; *Abcam*, #ab208572) or TRIS-EDTA Buffer (CD80; *Abcam*, #ab93684) at 100°C for 20min and 90°C for 20min. Endogenous peroxidase activity was quenched using BLOXALL blocking solution following the manufacturing protocol (*Vector Laboratories*, #SP-6000). Antibodies: anti-PD-L1 (*Abcam*, #ab205921; 1:100), anti-CD80 (*Abcam*, #ab254579; 1:100). The negative control slide underwent the same protocol as described above with the exception that no primary antibody was added. The supplied secondary antibody from the VectaStain Elite ABC Universal Plus kit was used at the recommended dilution.

Immunofluorescence

Tissues were prepared for immunofluorescence labeling as previously described(2, 4, 8) from 4µm sections with the following modification: Autofluorescence quenching with 0.1% Sudan Black in 70% ethanol, antigen retrieval using TRIS-EDTA Buffer (*Abcam*, #ab93684) at 100°C for 20min and 90°C for 20min, washes in TBST. Primary antibodies used were anti-mouse CD3e (*Abcam*, #ab11089; 1:100), anti-mouse E-cadherin (*BD Bioscience*, #610181; 1:50), and either anti-PD-1 (*Abcam*, #ab214421; 1:100), anti-PD-L1 (*Abcam*, #ab233482; 1:50), anti-CTLA-4 (*Abcam*, #ab237712; 1:100), or anti-CD80 (*Abcam*, #ab254579; 1:50). Secondary antibodies used were: goat anti-rat IgG H&L Alexa Fluor-594 (*Invitrogen*, #A-11007; 1:500), goat anti-mouse IgG2a Alexa Fluor-647 (*Invitrogen*, #A-21241; 1:500), goat anti-rabbit IgG H&L Alexa Fluor-488 (*Invitrogen*, #A-11008; 1:500). The slides were visualized using the Keyence BZ-X710.

Mouse Tissue Harvest

For all animal experiments, mice were euthanized by isoflurane exposure and cervical dislocation, and the body weight of each animal was recorded. Following, terminal heparin blood was collected via cardiac puncture. For mice analyzed by flow cytometry, mice were perfused with 20mL ice cold PBS/heparin (80units/mL) via pressure perfusion. After perfusion or directly after blood collection, the kidneys and spleen were harvested and weighed. For animals analyzed by flow cytometry, the left kidney and half the spleen were used for the single cell suspension/flow cytometry and half of the right kidney plus the other half of the spleen were fixed in 4% paraformaldehyde for histological analyses. The other half of the right kidney was flash frozen. For all other animals, the right kidney and half of the spleen were fixed in 4% paraformaldehyde for histological analyses and the left kidney plus the remainder of the spleen were flash frozen. The age of mice at euthanasia/tissue harvest varied depending on experiment (refer to results sections).

Histomorphometric and Kidney Function Analyses

Cystic index, cyst size, and cyst number were analyzed as previously published(4). In short, three kidney cross sections per animal (horizontal plane at pelvis, mid-superior and mid-inferior pole) were analyzed using a

custom-built NIS-Elements AR v4.6 macro (*Nikon*) - a cyst was defined as having a minimum feret diameter of 50µm.

Fibrotic area was analyzed from picrosirius red stained kidney sections visualized using an Olympus BX41 microscope with a linear polarizer as previously published(4). Ten random cortical 10x images were analyzed per animal.

Blood urea nitrogen (BUN) levels were analyzed using the terminal blood collection and following the manufacturer's protocol (QuantiChrom Urea Assay Kit, #501079333, *BioAssay Systems*). Samples were analyzed in duplicates.

Immunodepletion Experiments

Anti-PD-1 studies:

Four-month-old 129S6/SvEvTac *Pkd1*$^{RC/RC}$ mice were treated twice a week for eight weeks by intraperitoneal (IP) injection with 10mg/kg anti-PD-1 blocking antibody (clone RMP1-14; *Bio X Cell,* #BP0146) or 10mg/kg IgG2a control (clone 2A3; *Bio X Cell,* #BP-0089). After eight weeks of treatment, the animals were euthanized and evaluated as described above.

C57Bl/6J *Pkd1*$^{RC/-}$ mice were treated every other day by IP injection with 10mg/kg anti-PD-1 blocking antibody (clone RMP1-14; *Bio X Cell,* #BP0146) or 10 mg/kg IgG2a control (clone 2A3; *Bio X Cell,* #BP-0089) starting at postnatal day (P) 8 until P20, at which point the animals were euthanized and evaluated as described above. For both studies, the depletion antibody and the control antibody were diluted in PBS.

Anti-PD-1/Anti-CTLA-4 study:

One-month-old BALB/cJ *Pkd1*$^{RC/RC}$ mice were treated twice a week for eight weeks by IP injection with 10mg/kg anti-PD-1 blocking antibody (clone RMP1-14; *Bio X Cell,* #BP0146), 10mg/kg anti-CTLA-4 blocking antibody (clone 9D9; *Bio X Cell,* #BP0164), the combination of anti-PD-1 and anti-CTLA-4, or control IgG (10mg/kg IgG2a [clone 2A3; *Bio X Cell,* #BP0089] and 10mg/kg IgG2b control [clone MPC-11, *Bio X Cell,* #BP0086]). Animals with single blockade of PD-1 or CTLA-4 also received the respective other control antibody. Antibodies were diluted in InVivoPure pH 7.0 Dilution Buffer (*Bio X Cell,* #IP0070). After eight weeks of treatment, the animals were euthanized and evaluated as described above. **Supplemental Table 4** shows general

phenotyping characteristics of one-month-old BALB/cJ *Pkd1*$^{RC/RC}$ mice, providing a baseline of ADPKD severity at time of treatment start. These parameters were obtained from separate animals not included in the anti-PD-1/anti-CTLA-4 study.

The chosen dose of anti-PD-1 and anti-CTLA-4 is within the range that is clinically given, and the doses given as well as the frequency has been critically tested in multiple murine models(9-15).

Single Cell Suspension & Flow Cytometry

Single cell suspensions of the dissected kidneys were prepared as previously described(4, 8). In short, tissue was mechanically dissociated and digested in DMEM/F12 media (*Corning,* #MT15090CV) containing Liberase TL (2mg/mL, *Sigma-Aldrich*, #05401020001), and DNase I (20K U/mL, *Sigma-Aldrich,* #D5025) for 30min at 37°C. The digestion mix was passed through a 100µm and 70µm filter, as well as cleared of red blood cells using red blood cell lysis buffer (0.015M NH_4Cl, 10mM $KHCO_3$, 0.1mM Na_2EDTA, pH 7.2). Cells were pelleted and prepped for flow cytometry.

Staining of single cell suspension and flow cytometry protocol were described previously(4). Each kidney cell suspension was split in half and stained with two different panels (see below). The single cell suspension was blocked in anti-mouse CD16/CD32 (clone 93; *eBioscience,* #14-0161-86) for 15 min, followed by viability staining (LIVE/DEAD Fixable Aqua Dead Cell Stain Kit, *Invitrogen,* #L34966) for 15min and conjugated surface antibody staining for 30min. For the T cell panel outlined below, the single cell suspension then underwent fixation, permeabilization, and intracellular antibody staining according to the Foxp3/Transcription Factor Staining Buffer Set (*eBioscience*, #00-5523-00). All incubations were done at 4°C.

T cell Panel: CD44-FITC (clone IM7; 1:100; *eBioscience,* #11-0441-82), PD-1-PE (clone J43; 1:200; *eBioscience,* #12-9985-81), CD45-PE/CF594 (clone 30-F11; 1:100; *BD Biosciences,* #562420), TCRβ-PE/Cy5 (clone H57-597; 1:200; *eBioscience,* #15-5961-82), Ki-67-APC (clone SolA15; 1:400; *eBioscience,* #17-5698-82), CD69-PE-Cy7 (clone H1.2F3; 1:100; *eBioscience,* #25-0691-82), CD8-AF700 (clone 53-6.7; 1:100; *eBioscience,* #56-0081-82), CD4-APC/Cy7 (clone GK1.5; 1:200; *BioLegend,* #100414), FoxP3-eFluor450 (clone FJK-16s; 1:200; *eBioscience,* #48-5773-82).

Epithelial/macrophage panel: CD45-FITC (clone 30-F11; 1:100; *eBioscience,* #11-0451-85), CD11c-PE (clone HL3; 1:100; *BD Pharmingen,* #561044), CD19-PE/CF594 (clone 1D3; 1:100; *BD Horizen,* #562291); Ly6G-PercP/Cy5.5 (clone 1A8; 1:100; *Biolegend,* #127616), PD-L1-PE/Cy7 (clone MIH5; 1:100; *eBioscience,* #25-5982-82), CD64-AF647 (clone X54-5/7.1; 1:100' *BD Pharmingen,* #558539), APN-AF700 (clone ER-BMDM1; 1:100; *Novus Biologicals,* # NB10064843Z), EpCAM-APC/eFluor780 (clone G8.8; 1:100; *Invitrogen,* #47-5791-82).

Following staining cells were run on the Gallios Flow Cytometer Machine (*Beckman Coulter*) and analyzed using Kaluza Analysis v2.1 software (*Beckman Coulter*). For analyses, compensation was performed using single antibody-stained beads (VersaComp, #B22804, *Beckman Coulter*) and single antibody-stained cells. The gating strategy for the T cell panel was performed as previously published(4) with the following addition as outlined in(8): $CD8^+$ T cells were gated for staining positive for PD-1 or Ki-67. A fluorescence minus one (FMO) sample was used to set the negative gate.

Gating for PD-L1 positive epithelial cells and macrophages was done as follows and as outlined in(8): Live singlet cells were gated for CD45 positive or CD45 negative. CD45 negative cells were gated for being either APN^+ or $EpCAM^+$ using a Boolean "OR" gate (epithelial cells). Epithelial cells were then analyzed to be either positive or negative for PD-L1 staining. A FMO sample was used to set the negative gate (**Figure 1E**). Macrophages were defined by analyzing $CD45^+$ cells that were $CD19^-$ and $Ly6G^-$, to exclude B cells and neutrophils. Resulting cells were then gated for $CD64^+$ events, followed by analysis of PD-L1 positive expression using the same gate used for epithelial cells (**Figure 1C**).

For the CTLA-4 and CD80/CD86 analyses the following modifications were made to the protocol/panel: Epithelial/macrophages were stained using the following panel: CD45-FITC (clone 30-F11; 1:100; *eBioscience,* #11-0451-85), CD80-PE (clone 16-10A1; 1:100; *Biolegend,* #104708), CD19-PE/CF594 (clone 1D3; 1:100; *BD Horizen,* #562291); Ly6G-PercP/Cy5.5 (clone 1A8; 1:100; *Biolegend,* #127616), PD-L1-PE/Cy7 (clone MIH5; 1:100; *eBioscience,* #25-5982-82), CD64-AF647 (clone X54-5/7.1; 1:100; *BD Pharmingen,* #558539), APN-AF700 (clone ER-BMDM1; 1:100; *Novus Biologicals,* # NB10064843Z), EpCAM-APC/eFluor780 (clone G8.8; 1:100; *Invitrogen,* #47-5791-82), CD86-PacBlue (clone GL-1; 1:300; *Biolegend*, #105012).

T-cell CTLA-4 expression was detected as follows: Pelleted cells were suspended in 2 mL of R10 buffer comprised of RPMI 1640 with L-glutamine (*Corning*, #10-040-CV), 10% FBS (*Sigma-Aldrich*, #MFCD00132239), and 10 mM HEPES (*Corning*, #25-060-CI). Cell Stimulation Cocktail (*eBioscience*, #00-4970-03) containing phorbol 12-myristate 13-acetate and ionomycin was added at 1X and cells were incubated for one hour at 37°C. Without washing, BD GolgiStop (*BD Biosciences*, #BDB554724) protein transport inhibitor containing monensin was added at 6.67 μL/mL R10 buffer, and cells were incubated at 37°C for an additional 4 hours. After 2 washes with FA3 Buffer, cells were blocked with anti-mouse CD16/CD32, viability stained, and surface stained with the following antibodies: anti-CD45-AF532 (clone: 30-F11; 1:200; *eBioscience*, #58-0451-80), anti-MHCII-BUV661 (clone: M5/114.15.2; 1:2000; *BD Biosciences*, #750280), anti-TCRβ-BUV615 (clone: H57-597; 1:100; *BD Biosciences*, #751212), anti-CD8-BV570 (clone: 53-6.7; 1:100, *Biolegend*, #100739), anti-CD4-PacificBlue (clone: RM4-5; 1:200; *Biolegend*, #100534), anti-CD25-BB515 (clone: PC61; 1:400; *eBioscience*, #564458), anti-PD-1-BUV737 (clone: RMP1-30; 1:100, *BD Biosciences*, #749306), anti-CTLA-4-APC (clone: UC10-B49; 1:200; *Biolegend*, #106309). Cells were then fixed and permeabilized according to the Cyto-Fast Fix/Perm Buffer Set (*BioLegend*, #426803). The next day, anti-CLTA-4 antibody was applied to stain intracellular CTLA-4 for 1h. Cells were washed as described above and ran on the Cytek® Aurora spectral flow cytometer.

Statistics

All analyses were performed using JMP Pro 16.1 (*SAS*) or PRISM 9.2.0 (*Graphpad Software*). Data are presented as the mean ± standard error of the mean (SEM) or box plot (25th to 75th percentile and median) with whiskers of 10-90th percentile; single data points are depicted. Pairwise comparisons were performed using a Mann-Whitney test and multi-group comparisons were performed using Kruskal-Wallis test or Brown-Forsythe test (if N<4) with multiple comparison follow-up by controlling for FDR (Benjamini, Krieger, Yekutieli). The Pearson correlation coefficient was computed using two-tailed multivariate analyses with a confidence interval of 95%. P-values are denoted by *(p<0.05), **(p<0.01), ***(p<0.001), and ****(p<0.0001).

Study Approval

All experimental procedures were performed in an AAALAC-accredited facility in accordance with the *Guide for the Care and Use of Laboratory Animals*(16) and approved by the University of Colorado Anschutz Medical Campus Institutional Animal Care and Use Committee (protocols #33, 301, 685).

Data Availability

All underlying data generated as part of this manuscript are available from the corresponding author upon request and an MTA in place.

SUPPLEMENTAL TABLES

Supplemental Table 1 | Population specific distribution of PD-1/PD-L1 expressing cells in mouse wildtype and ADPKD1 kidneys.

	$CD8^+$, $PD\text{-}1^+$ (%$CD8^+$)			$CD64^+$, $PD\text{-}L1^+$ (%$CD64^+$)			$EpCAM^+/APN^+$, $PD\text{-}L1^+$ (%$EpCAM^+/APN^+$)		
C57Bl/6J	WT	*Pkd1* $^{RC/RC}$	*Statistics*	WT	*Pkd1* $^{RC/RC}$	*Statistics*	WT	*Pkd1* $^{RC/RC}$	*Statistics*
3mo	12.88±2.79	13.76±2.11	ns	1.30±0.10	1.26±0.23	ns	1.78±0.23	1.93±0.16	ns
6mo	28.92±2.98	28.72±4.08	ns/##	1.82±0.32	2.13±0.30	ns/#	2.25±0.21	3.55±0.39	**/###
9mo	37.54±3.56	31.80±2.97	ns/###/ns	4.48±0.66	4.67±0.55	ns/####/^^	2.05±0.32	7.96±1.74	****/####/^
129S6/SvEVTac	WT	*Pkd1* $^{RC/RC}$	*Statistics*	WT	*Pkd1* $^{RC/RC}$	*Statistics*	WT	*Pkd1* $^{RC/RC}$	*Statistics*
3mo	7.48±1.69	9.36±0.94	ns	0.94±0.18	2.28±0.24	**	0.72±0.05	3.81±0.60	***
6mo	4.27±1.06	14.02±2.27	**/ns	1.29±0.17	2.70±0.32	**/ns	1.57±0.13	6.13±0.96	***/ns
9mo	1.56±0.74	7.05±1.23	**/ns/^	0.91±0.12	1.92±0.20	**/ns/ns	1.04±0.13	4.62±0.59	**/ns/ns
BALB/cJ	WT	*Pkd1* $^{RC/RC}$	*Statistics*	WT	*Pkd1* $^{RC/RC}$	*Statistics*	WT	*Pkd1* $^{RC/RC}$	*Statistics*
3mo	0.35±0.12	9.28±0.98	**	1.23±0.06	1.76±0.24	ns	0.78±0.07	1.92±0.24	***
6mo	2.65±0.32	26.92±3.49	**/###	1.19±0.13	2.25±0.41	**/ns	1.06±0.08	4.68±0.31	**/###
9mo	8.59±4.07	25.15±2.17	*/###/ns	4.45±1.93	3.36±0.35	ns/##/^	2.25±0.65	7.07±0.69	**/###/^

mean±SEM; Statistic: *,^ <0.05; **, ##,^^ <0.01; ***, ### <0.001; ****, #### <0.0001, ns: non-significant

Statistic: WT vs. *Pkd1* $^{RC/RC}$ (star, *); *Pkd1* $^{RC/RC}$ 3mo vs. 6mo or 3mo vs. 9mo (number sign, #); *Pkd1* $^{RC/RC}$ 6mo vs. 9mo (circumflex, ^)

Supplemental Table 2 | Analysis of the macrophage and epithelial cell population in mouse wildtype and ADPKD1 kidneys.

	$CD64^+$ (%live)			$APN^+/EpCAM^+$ (%live)		
C57Bl/6J	WT	*Pkd1* $^{RC/RC}$	*Statistics*	WT	*Pkd1* $^{RC/RC}$	*Statistics*
3mo	1.75±0.17	2.16±0.23	ns	85.93±1.73	89.12±0.79	ns
6mo	2.93±0.44	4.54±0.36	ns/####	77.43±2.47	74.99±2.04	ns/####
9mo	1.99±0.21	4.57±0.82	*/ns/ns	84.93±1.55	67.30±2.55	****/####/^
129S6/SvEVTac	WT	*Pkd1* $^{RC/RC}$	*Statistics*	WT	*Pkd1* $^{RC/RC}$	*Statistics*
3mo	0.88±0.08	2.68±0.42	***	89.45±0.73	83.97±1.50	**
6mo	1.23±0.19	3.70±0.45	***/ns	88.34±1.20	85.89±1.06	ns/ns
9mo	2.09±0.45	7.12±0.79	**/###/^^	86.97±0.94	73.44±1.90	**/##/^^
BALB/cJ	WT	*Pkd1* $^{RC/RC}$	*Statistics*	WT	*Pkd1* $^{RC/RC}$	*Statistics*
3mo	0.55±0.10	3.86±0.53	***	93.70±0.67	87.68±1.26	**
6mo	0.70±0.09	3.56±0.71	***/ns	89.68±1.21	81.35±2.29	*/ns
9mo	1.28±0.41	3.10±0.49	*/ns/ns	88.48±3.08	71.40±7.28	ns/#/ns

mean±SEM; Statistic: *,^ <0.05; **, ##,^^ <0.01; ***, ### <0.001; ****, #### <0.0001, ns: non-significant

Statistic: WT vs. *Pkd1* $^{RC/RC}$ (star, *); *Pkd1* $^{RC/RC}$ 3mo vs. 6mo or 3mo vs. 9mo (number sign, #); *Pkd1* $^{RC/RC}$ 6mo vs. 9mo (circumflex, ^)

Supplemental Table 3 | PKD histomorphometric analyses of various ADPKD1 models with either genetic *Pd-l1* loss or anti-PD-1 treatment.

	Cyst Size (μm^2)	Cyst Number/mm^2	Fibrotic Index (%)
C57Bl/6J *Pkd1* $^{RC/RC}$			
Cd274 $^{+/+}$	21,775±2,310	15.49±1.45	14.60±2.25
Cd274 $^{-/-}$	18,891±1,289	16.53±1.35	12.70±1.20
BALB/cJ *Pkd1* $^{RC/RC}$			
Cd274 $^{+/+}$	30,496±5,502	12.77±1.086	14.32±1.02
Cd274 $^{-/-}$	29,404±5,926	13.32±0.75	11.75±2.11
129S6/SvEVTac *Pkd1* $^{RC/RC}$			
IgG2a	64,099±8,355	9.02±0.75	17.03±0.74
α-PD-1	57,198±5,850	9.00±0.66	15.47±1.45
C57Bl/6J *Pkd1* $^{RC/-}$			
IgG2a	136,407±16,515	6.27±0.50	26.64±3.52
α-PD-1	161,353±23,387	6.10±1.24	26.52±2.87

mean±SEM; Mann-Whitney Test for all pairwise comparisons was non-significant.

Supplemental Table 4 | Baseline PKD phenotyping characteristics of 1-month-old BALB/cJ *Pkd1*$^{RC/RC}$ mice. Separate animals not included in the combination immune checkpoint blockade trial were analyzed.

	**1-month-old BALB/cJ *Pkd1* $^{RC/RC}$**
KW/BW (%)	3.21±0.27
Cystic Index (%)	21.96±2.38
Cyst Size (μm^2)	15,397±1,822
Cyst Number/mm^2	14.87±1.14
Fibrotic Index (%)	4.98±0.71
BUN (mg/dL)	27.72±0.76

mean±SEM, N=2 males, 2 females

Supplemental Table 5 | Correlation data of adaptive immune cell phenotypes with PKD parameters in 3-month-old BALB/cJ *Pkd1*$^{RC/RC}$ mice part of the combination immune checkpoint blockade trial.

	%KW/BW	Cystic Index	Fibrotic Index
CD8$^+$ (%CD45)			
Pearson *r*	-0.181	-0.170	-0.261
P value	0.358	0.387	0.179
CD4$^+$ (%CD45)			
Pearson *r*	-0.300	-0.275	-0.358
P value	0.121	0.157	0.061
CD4$^+$, FoxP3$^+$ (%CD45)			
Pearson *r*	0.204	0.127	0.178
P value	0.299	0.521	0.365
**CD8$^+$, CD44$^+$/CD69$^+$ (%CD45)**			
Pearson *r*	-0.387	-0.402	-0.436
P value	**0.042**	**0.034**	**0.020**
CD8$^+$, Ki67$^+$ (%CD45)			
Pearson *r*	-0.373	-0.315	-0.342
P value	0.051	0.103	0.075
CD4$^+$, CD44$^+$/CD69$^+$ (%CD45)			
Pearson *r*	-0.374	-0.385	-0.374
P value	0.050	**0.043**	0.050
CD4$^+$, Ki67$^+$ (%CD45)			
Pearson *r*	-0.249	-0.310	-0.359
P value	0.202	0.109	0.061
**CD8$^+$, CD44$^+$/CD69$^+$ or Ki67$^+$ (%CD45)**			
Pearson *r*	-0.400	-0.390	-0.424
P value	**0.035**	**0.040**	**0.025**
CD4$^+$, CD44$^+$/CD69$^+$ or Ki67$^+$ (%CD45)			
Pearson *r*	-0.365	-0.382	-0.378
P value	0.056	**0.045**	**0.047**
**CD8$^+$ and CD4$^+$, CD44$^+$/CD69$^+$ (%CD45)**			
Pearson *r*	-0.381	-0.393	-0.386
P value	**0.046**	**0.039**	**0.042**
CD8$^+$ and CD4$^+$, Ki67$^+$ (%CD45)			
Pearson *r*	-0.311	-0.335	-0.381
P value	0.108	0.081	**0.046**
**CD8$^+$ and CD4$^+$, CD44$^+$/CD69$^+$ or Ki67$^+$ (%CD45)**			
Pearson *r*	-0.377	-0.391	-0.392
P value	**0.048**	**0.040**	**0.039**
**CD8$^+$, CD44$^+$/CD69$^+$ or Ki67$^+$ (%CD45) (positive variable); CD4+, FoxP3+ (%CD45) (negative variable)**			
Pearson *r*	-0.456	-0.371	-0.445
P value	**0.015**	**0.052**	**0.018**
**CD8$^+$and CD4$^+$, CD44$^+$/CD69$^+$ or Ki67$^+$ (%CD45) (positive variable); CD4+, FoxP3+ (%CD45) (negative variable)**			
Pearson *r*	-0.462	-0.459	-0.472
P value	**0.013**	**0.014**	**0.011**

Values in boldface font style are statistically significant.

Supplemental Table 6 | Analysis of PD-1 expression on $CD4^+$ T cells in mouse wildtype and ADPKD1 kidneys.

	$CD4^+$, PD-1$^+$ (%live)			$CD4^+$, PD-1$^+$ (%$CD4^+$)		
C57Bl/6J	WT	*Pkd1* $^{RC/RC}$	*Statistics*	WT	*Pkd1* $^{RC/RC}$	*Statistics*
3mo	0.0065±0.0014	0.051±0.0082	***	5.12±0.83	16.81±1.83	***
6mo	0.015±0.0024	0.10±0.020	****/ns	11.53±1.43	25.51±2.38	***/#
9mo	0.012±0.0012	0.32±0.088	****/##/^	10.50±1.17	23.81±1.96	****/#/ns
129S6/SvEVTac	WT	*Pkd1* $^{RC/RC}$	*Statistics*	WT	*Pkd1* $^{RC/RC}$	*Statistics*
3mo	0.0065±0.0011	0.12±0.027	***	10.10±1.35	27.26±1.26	***
6mo	0.0044±0.0012	0.38±0.062	***/##	3.89±0.78	28.56±2.38	***/ns
9mo	0.044±0.026	1.30±0.39	**/###/^	3.38±0.90	17.05±1.03	**/###/^^
BALB/cJ	WT	*Pkd1* $^{RC/RC}$	*Statistics*	WT	*Pkd1* $^{RC/RC}$	*Statistics*
3mo	0.0014±0.00071	0.17±0.016	***	1.22±0.48	29.18±1.14	***
6mo	0.0015±0.00029	0.28±0.029	**/##	1.78±0.64	25.28±3.65	**/ns
9mo	0.017±0.0095	0.38±0.055	**/##/ns	6.50±2.14	26.02±2.88	**/ns/ns

mean±SEM; Statistic: *,^ <0.05; **, ##,^^ <0.01; ***, ### <0.001; ****, #### <0.0001, ns: non-significant

Statistic: WT vs. *Pkd1* $^{RC/RC}$ (star, *); *Pkd1* $^{RC/RC}$ 3mo vs. 6mo or 3mo vs. 9mo (number sign, #); *Pkd1* $^{RC/RC}$ 6mo vs. 9mo (circumflex, ^)

SUPPLEMENTAL FIGURES

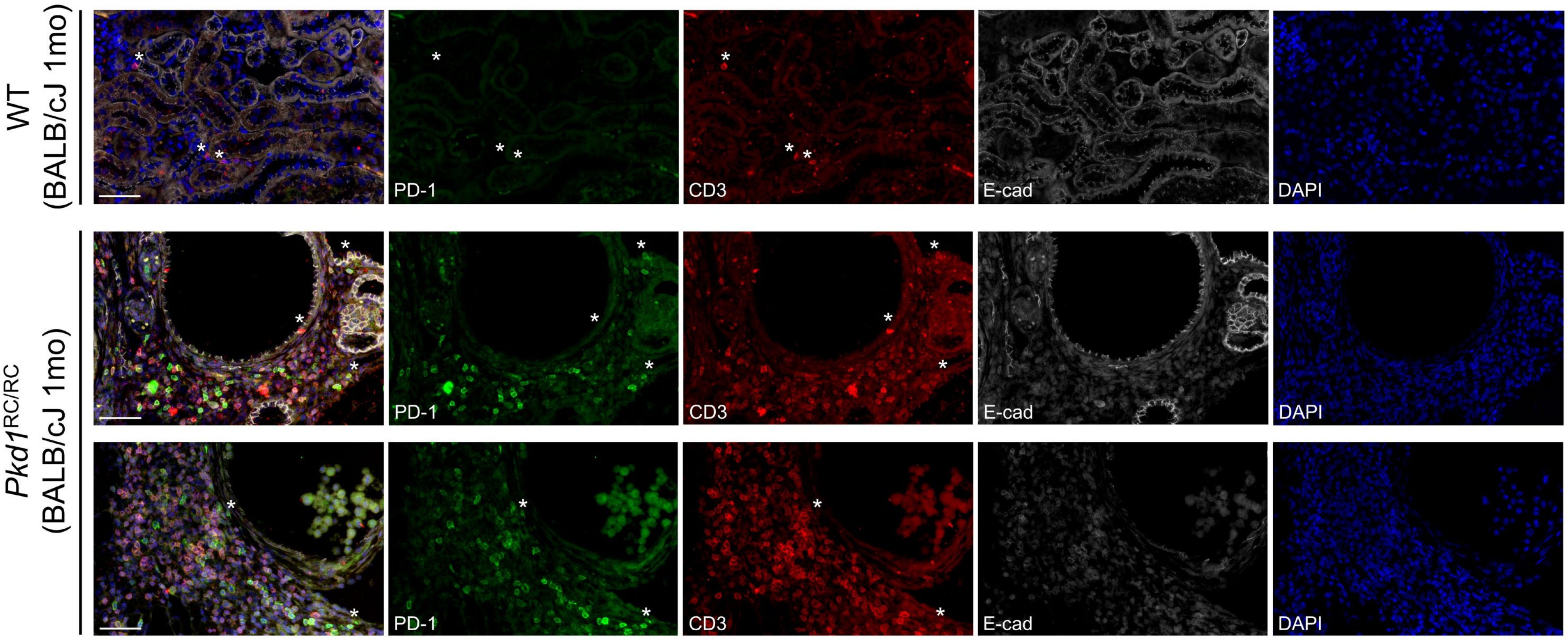

Supplemental Figure 1 | T cells stain positive for PD-1 in *Pkd1*$^{RC/RC}$ but not wildtype kidneys.

BALB/cJ wildtype (WT) and *Pkd1*$^{RC/RC}$ kidneys were stained for PD-1 by immunofluorescent labeling. Two different *Pkd1*$^{RC/RC}$ mice were used and representative images for each genotype are shown; merged (far left) followed by each single channel. Note: anti-CD3, conjugated to AF594, and anti-E-cadherin, conjugated to AF647, have some spectral bleed-through. In *Pkd1*$^{RC/RC}$ but not WT kidneys T cells stain positive for PD-1 and are near cystic lesions. *WT: T cells; *Pkd1*$^{RC/RC}$: PD-1 positive T cells that are in direct contact with epithelial cells. Scale bar: 50µm. Green = PD-1; red = CD3; white = E-cadherin, blue = DAPI; mo=month.

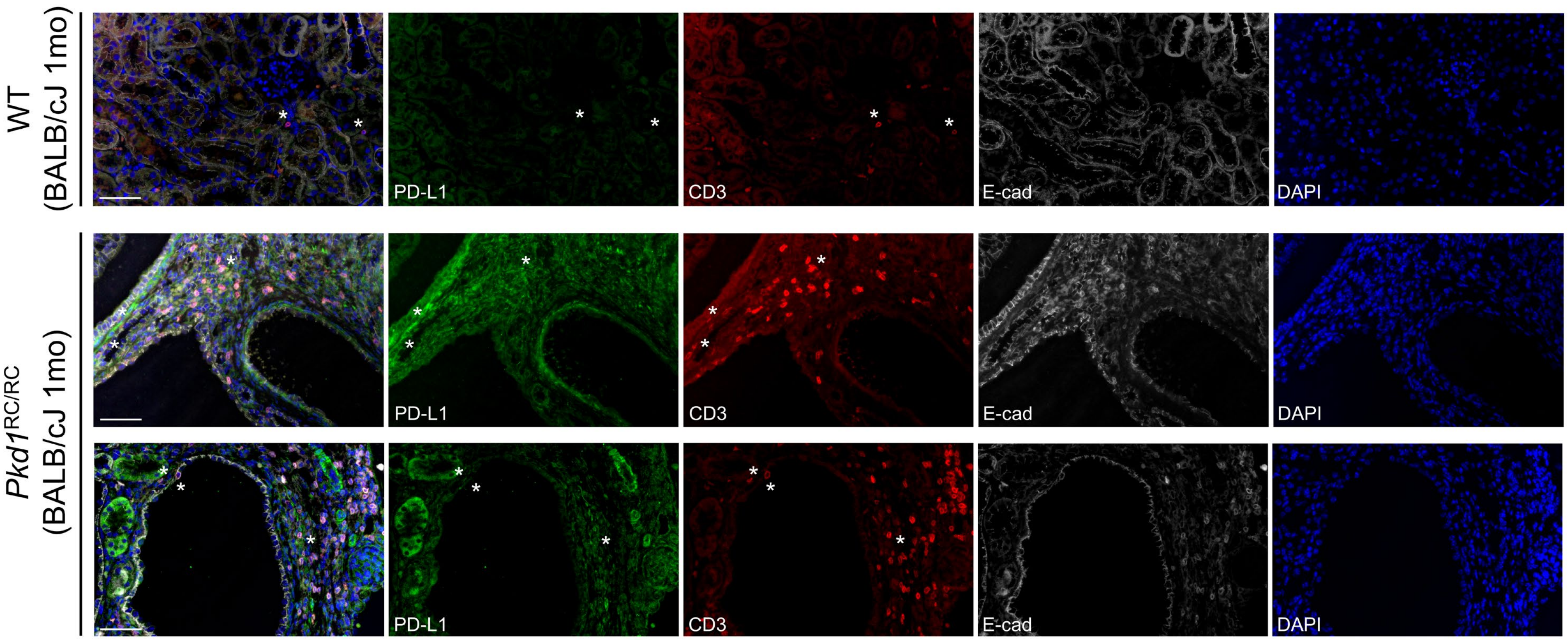

Supplemental Figure 2 | Interstitial and epithelial cells stain positive for PD-L1 in *Pkd1*$^{RC/RC}$ but not wildtype kidneys.

BALB/cJ wildtype (WT) and *Pkd1*$^{RC/RC}$ kidneys were stained for PD-L1 by immunofluorescent labeling. Two different *Pkd1*$^{RC/RC}$ mice were used and representative images for each genotype are shown; merged (far left) followed by each single channel. Note: anti-CD3, conjugated to AF594, and anti-E-cadherin, conjugated to AF647, have some spectral bleed-through. In cystic regions of *Pkd1*$^{RC/RC}$ kidneys but not in WT kidneys interstitial and tubular epithelial cells stain positive for PD-L1. *WT: T cells; *Pkd1*$^{RC/RC}$: PD-L1 positive interstitial or epithelial cells in direct contact with T cells. Scale bar: 50μm. Green = PD-L1; red = CD3; white = E-cadherin, blue = DAPI; mo=month.

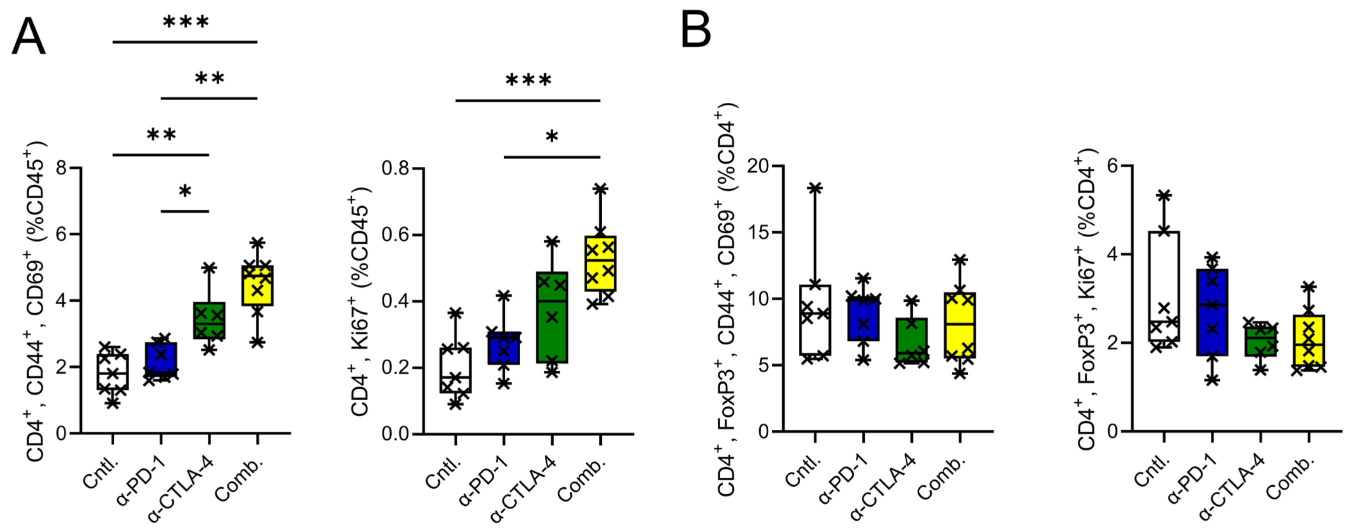

Supplemental Figure 3 | Overall CD4$^+$ T cells but not the T$_{Reg}$ subset show increased activation and proliferation upon combination immune checkpoint blockade.

Flow cytometry of kidney single cell suspensions obtained at the end of treatment with either α-PD-1 (blue), α-CTLA-4 (green), or the combination (Comb., yellow) in BALB/cJ *Pkd1*$^{RC/RC}$ mice compared to control (Cntl., white). Number of kidney CD4$^+$ T cells (**A**) or CD4$^+$;FoxP3$^+$ cells (T$_{Regs}$, **B**) expressing CD44 and CD69, markers of T cell activation, or Ki67, marker of proliferation as percent CD45$^+$ (**A**) or CD4$^+$ (**B**). Monotherapy with either α-PD-1 or α-CTLA-4 increased CD4$^+$ T cell numbers, activation, and proliferation; however, the effect was amplified by combination treatment. Neither single nor combination immune checkpoint blockade impacted the activation or proliferation status of T$_{Regs}$. Data are presented as box plot (25th to 75th percentile and median) with whiskers of 10-90th percentile; single data points are depicted. Kruskal-Wallis one-way analysis of variance with multiple comparison follow-up by controlling for false discovery rate (Benjamini, Krieger, Yekutieli) were performed. *p<0.05, **p<0.01, ***p<0.001. N=7-8 mice per group. Non-significant pairwise comparisons are not shown.

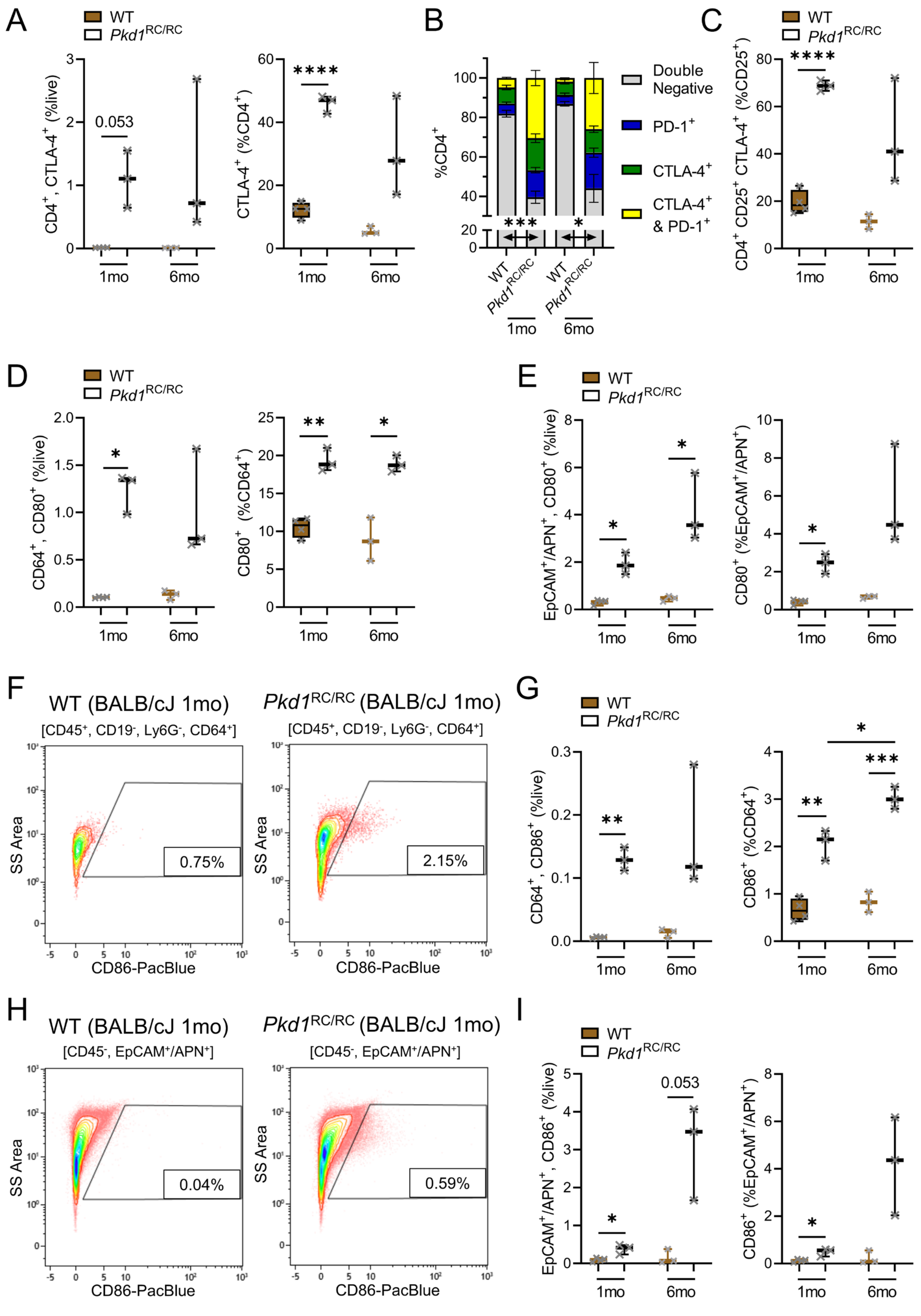

Supplemental Figure 4 | CTLA-4 is upregulated on CD4⁺ T cells and T$_{Regs}$, as well is CD80 and CD86 on macrophages and epithelial cells.

Kidneys of BALB/cJ wildtype (WT) and *Pkd1*$^{RC/RC}$ mice were harvested at 1 and 6 months (mo) of age and analyzed by flow cytometry to identify the expression of CTLA-4 on $CD4^+$ T cells ($CD45^+$/$TCR\beta^+$/$CD4^+$; **A-C**) and CD80 (**D, E**) or CD86 on macrophages ($CD45^+$/$CD19^-$/$Ly6G^-$/$CD64^+$ [**F, G**]) or on epithelial cells ($CD45^-$/$EpCAM^+$/APN^+ [**H, I**]). (**F, H**) Representative flow diagrams of 1-month-old BALB/cJ WT and *Pkd1*$^{RC/RC}$ kidneys showing positive CD86 populations (see **Figure 6** for CD80). Quantification of CTLA-4 positive $CD4^+$ T cells (**A**), CD80 or CD86 positive $CD64^+$ macrophages (**D, G**), and CD80 or CD86 positive epithelial cells (**E, I**) as percent live (%live) and % parent population comparing WT (brown) to *Pkd1*$^{RC/RC}$ (white) kidneys. (**C**) Quantification of CTLA-4 positive T_{Regs} as percent of total T_{Regs} comparing WT (brown) to *Pkd1*$^{RC/RC}$ (white) kidneys. CTLA-4 or CD80/CD86 expression is significantly upregulated on the respective cell type in *Pkd1*$^{RC/RC}$ compared to WT kidneys. (**B**) Analysis of $CD4^+$ T cells that express either PD-1 (blue), CTLA-4 (green), PD-1 and CTLA-4 (yellow), or none of the immune checkpoint receptors (grey). Compared to $CD8^+$ T cells (**Figure 6G**) there is a large portion of $CD4^+$ T cells that co-express PD-1 and CTLA-4; but there are also sufficient numbers of $CD4^+$ T cells that express only one of the immune checkpoint receptors. (**A, C, D, E, G, I**) Data are presented as box plot (25th to 75th percentile and median) with whiskers of 10-90th percentile; single data points are depicted. Brown-Forsythe one-way analysis of variance with multiple comparison follow-up by controlling for false discovery rate (Benjamini, Krieger, Yekutieli) were performed. Non-significant pair-wise comparisons are not shown. (**B**) Comparisons shown are limited to cells expressing neither immune checkpoint protein (grey). *$p<0.05$; **$p<0.01$; ***$p<0.001$; ****$p<0.0001$; N=4 for BALB/cJ WT and N=3 BALB/cJ *Pkd1*$^{RC/RC}$. Data points are male and female.

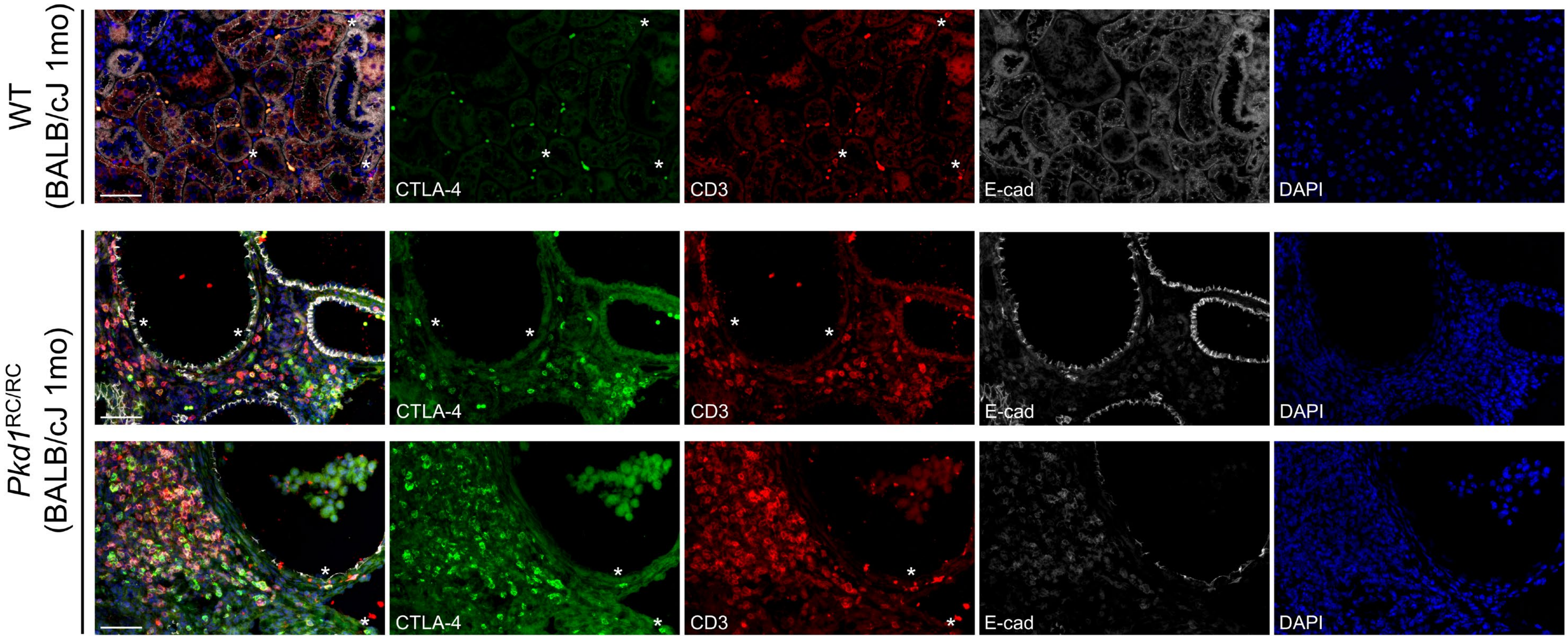

Supplemental Figure 5 | T cells stain positive for CTLA-4 in *Pkd1*$^{RC/RC}$ but not wildtype kidneys.

BALB/cJ wildtype (WT) and *Pkd1*$^{RC/RC}$ kidneys were stained for CTLA-4 by immunofluorescent labeling. Two different *Pkd1*$^{RC/RC}$ mice were used and representative images for each genotype are shown; merged (far left) followed by each single channel. Note: anti-CD3, conjugated to AF594, and anti-E-cadherin, conjugated to AF647, have some spectral bleed-through. In *Pkd1*$^{RC/RC}$ but not WT kidneys T cells stain positive for CTLA-4 and are near cystic lesions. *WT: T cells; *Pkd1*$^{RC/RC}$: CTLA-4 positive T cells that are in direct contact with epithelial cells. *Pkd1*$^{RC/RC:}$ Serial sections were stained for PD-1 and CTLA-4 (**Supplemental Figure 1**) allowing comparison of T cells positive for either PD-1 or CTLA-4. Scale bar: 50µm. Green = CTLA-4; red = CD3; white = E-cadherin, blue = DAPI; mo=month.

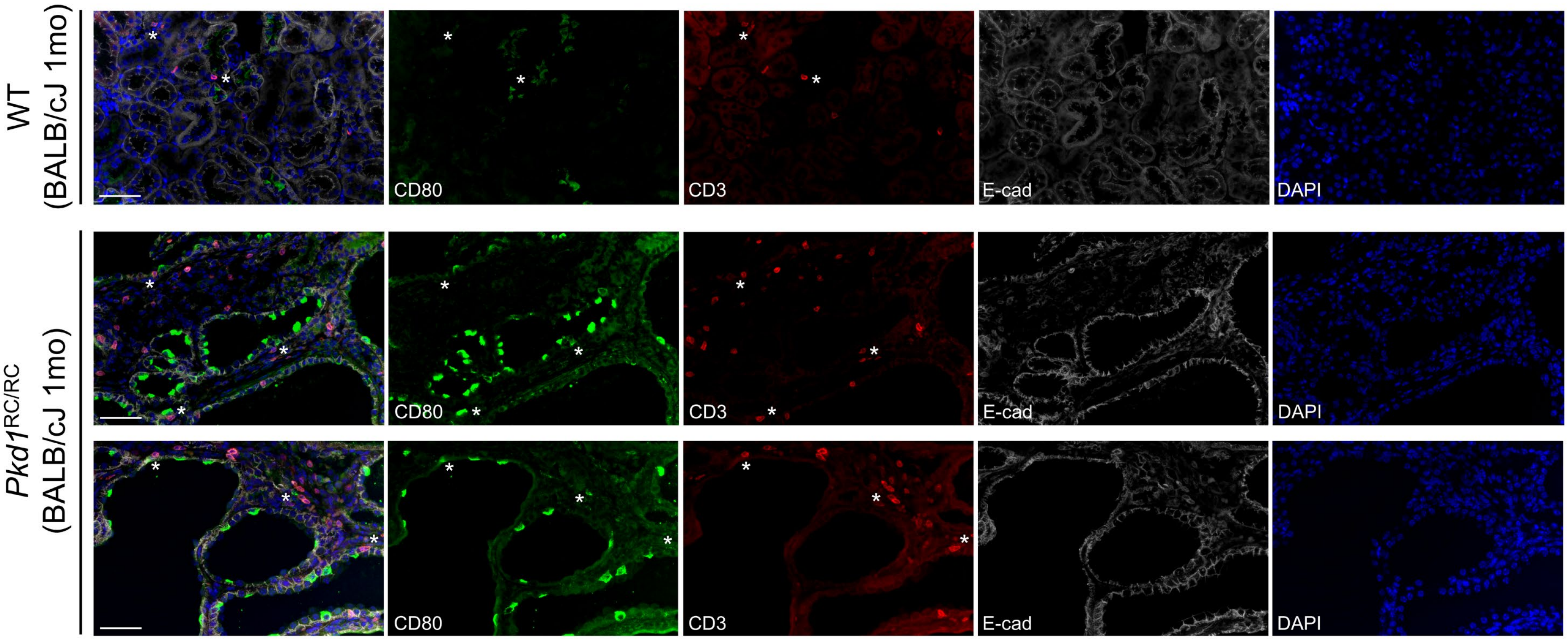

Supplemental Figure 6 | Epithelial cells stain positive for CD80 in *Pkd1*$^{RC/RC}$ but not wildtype kidneys.

BALB/cJ wildtype (WT) and *Pkd1*$^{RC/RC}$ kidneys were stained for CD80 by immunofluorescent labeling. Two different *Pkd1*$^{RC/RC}$ mice were used and representative images for each genotype are shown; merged (far left) followed by each single channel. Note: anti-CD3, conjugated to AF594, and anti-E-cadherin, conjugated to AF647, have some spectral bleed-through. In cystic regions of *Pkd1*$^{RC/RC}$ kidneys but not in WT kidneys tubular epithelial cells stain strongly positive for CD80. *WT: T cells; *Pkd1*$^{RC/RC}$: CD80 positive epithelial or interstitial cells in direct contact with T cells. Scale bar: 50µm. Green = CD-80; red = CD3; white = E-cadherin, blue = DAPI; mo=month.

SUPPLEMENTAL REFERENCES

1. Arroyo J, Escobar-Zarate D, Wells HH, Constans MM, Thao K, Smith JM, et al. The genetic background significantly impacts the severity of kidney cystic disease in the Pkd1(RC/RC) mouse model of autosomal dominant polycystic kidney disease. *Kidney Int.* 2021;99(6):1392-407.
2. Hopp K, Ward CJ, Hommerding CJ, Nasr SH, Tuan HF, Gainullin VG, et al. Functional polycystin-1 dosage governs autosomal dominant polycystic kidney disease severity. *J Clin Invest.* 2012;122(11):4257-73.
3. Hopp K, Hommerding CJ, Wang X, Ye H, Harris PC, and Torres VE. Tolvaptan plus pasireotide shows enhanced efficacy in a PKD1 model. *J Am Soc Nephrol.* 2015;26(1):39-47.
4. Kleczko EK, Marsh KH, Tyler LC, Furgeson SB, Bullock BL, Altmann CJ, et al. CD8(+) T cells modulate autosomal dominant polycystic kidney disease progression. *Kidney Int.* 2018;94(6):1127-40.
5. Muto S, Aiba A, Saito Y, Nakao K, Nakamura K, Tomita K, et al. Pioglitazone improves the phenotype and molecular defects of a targeted Pkd1 mutant. *Hum Mol Genet.* 2002;11(15):1731-42.
6. Dong H, Zhu G, Tamada K, Flies DB, van Deursen JM, and Chen L. B7-H1 determines accumulation and deletion of intrahepatic CD8(+) T lymphocytes. *Immunity.* 2004;20(3):327-36.
7. Loghman-Adham M, Nauli SM, Soto CE, Kariuki B, and Zhou J. Immortalized epithelial cells from human autosomal dominant polycystic kidney cysts. *Am J Physiol Renal Physiol.* 2003;285(3):F397-412.
8. Nguyen DT, Kleczko EK, Dwivedi N, Monaghan MT, Gitomer BY, Chonchol MB, et al. The tryptophan-metabolizing enzyme indoleamine 2,3-dioxygenase 1 regulates polycystic kidney disease progression. *J Clin Invest Insight.* 2023;8(1).
9. da Fonseca-Martins AM, Ramos TD, Pratti JES, Firmino-Cruz L, Gomes DCO, Soong L, et al. Immunotherapy using anti-PD-1 and anti-PD-L1 in Leishmania amazonensis-infected BALB/c mice reduce parasite load. *Sci Rep.* 2019;9(1):20275.
10. Johnson AM, Bullock BL, Neuwelt AJ, Poczobutt JM, Kaspar RE, Li HY, et al. Cancer Cell-Intrinsic Expression of MHC Class II Regulates the Immune Microenvironment and Response to Anti-PD-1 Therapy in Lung Adenocarcinoma. *J Immunol.* 2020;204(8):2295-307.

11. Selby MJ, Engelhardt JJ, Johnston RJ, Lu LS, Han M, Thudium K, et al. Preclinical Development of Ipilimumab and Nivolumab Combination Immunotherapy: Mouse Tumor Models, In Vitro Functional Studies, and Cynomolgus Macaque Toxicology. *PLoS One.* 2016;11(9):e0161779.

12. George S, Motzer RJ, Hammers HJ, Redman BG, Kuzel TM, Tykodi SS, et al. Safety and Efficacy of Nivolumab in Patients With Metastatic Renal Cell Carcinoma Treated Beyond Progression: A Subgroup Analysis of a Randomized Clinical Trial. *JAMA Oncol.* 2016;2(9):1179-86.

13. Philips GK, and Atkins M. Therapeutic uses of anti-PD-1 and anti-PD-L1 antibodies. *Int Immunol.* 2015;27(1):39-46.

14. Renner A, Burotto M, and Rojas C. Immune Checkpoint Inhibitor Dosing: Can We Go Lower Without Compromising Clinical Efficacy? *J Glob Oncol.* 2019;5:1-5.

15. Calabro L, Morra A, Fonsatti E, Cutaia O, Fazio C, Annesi D, et al. Efficacy and safety of an intensified schedule of tremelimumab for chemotherapy-resistant malignant mesothelioma: an open-label, single-arm, phase 2 study. *Lancet Respir Med.* 2015;3(4):301-9.

16. Research IoLA. *Guide for the Care and Use of Laboratory Animals.* Washington (DC): National Academies Press; 2011.
